# A Novel Microfluidic‐Based Fluorescence Detection Method Reveals Heavy Atom Effects on Photophysics of Fluorophores With High Triplet Quantum Yield: A Numerical Simulation Study

**DOI:** 10.1002/bio.70090

**Published:** 2025-01-20

**Authors:** Selim Can Dirican, Barış Demirbay

**Affiliations:** ^1^ Department of Mechanical Engineering Özyeğin University Istanbul Türkiye; ^2^ Department of Mathematical and Natural Sciences Özyeğin University Istanbul Türkiye

**Keywords:** fluorescence spectroscopy, fluorophore blinking, heavy atom effect, microfluidics, photophysics, widefield microscopy

## Abstract

The present study introduces the idea of a novel fluorescence‐based imaging technique combined with a microfluidic platform that enables a precise control of dark transient state populations of fluorescent probes flowing over a uniform, top flat supergaussian excitation field with a constant flow rate. To demonstrate the imaging capability of the proposed detection method, numerical simulations have been performed by considering laser, microscope and flow parameters of experimental setup together with photophysical model and electronic transition rates of fluorescent dyes. As an output data to be assessed, fluorescence image data is simulated numerically for bromine‐free carboxyfluorescein and its brominated derivatives having different numbers of bromine atoms. Based on the magnitudes of applied excitation irradiances and flow rates, which can be manually controlled by user during experiments, the presence of dark state populations can appear as broadening, shifts and decays in normalized fluorescence intensity signals that are computed from simulated fluorescence images. As such changes in signals become more pronounced upon an increase in the degree of bromination, it is elicited that heavy atom effect can be resolved by properly tuning excitation powers of laser and flow rates. Proposed imaging method has potential to provide invaluable means to conventional fluorescence methods and can open up new perspectives in biomedical research.

## Graphical abstract

Numerical simulations adapted for a proposed microfluidics‐based widefield fluorescence imaging technique reveal controllability of long‐lived, dark transient state build‐ups in ${\left\langle F\right\rangle }_{\mathrm{norm}}$ signals of brominated carboxyfluorescein derivatives when flowing over a uniform, flattop supergaussian excitation field with constant flow rates under a continuous laser excitation.

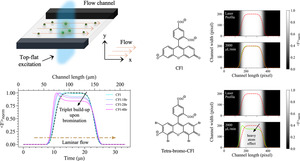

## 1 Introduction

In recent years, there have been significant advancements in the sensitivity, specificity, and spatial resolution of fluorescence‐based spectroscopy and imaging techniques [1, 2]. Such methodological developments, to a large extent, are reliant on blinking properties of fluorescent probes. Photoinduced dark transient states have thus been particularly invaluable in revealing how these fluorescent markers can undergo reactions and interact with a complex cellular environment at the molecular level, as well as enhancing resolution in fluorescence‐based microscopy and spectroscopy [3, 4]. In this respect, population dynamics of long‐lived, nonfluorescent or weakly fluorescent transient states of fluorophores arising from intersystem crossing (ISC) to triplet states and photoinduced charge transfer processes can offer useful information and be greatly exploited as read‐out parameters in biomolecular studies [5, 6]. Particularly, fluorescent probes with high ISC have a unique property that enables them to efficiently transfer excited electrons from their excited singlet state to the triplet state, resulting in long‐lived and highly emissive triplet states [7].

Having dark state transitions makes such fluorescent markers very useful in multitude of biomedical applications including photodynamic therapy (PDT) [8], biosensing [9] and imaging [10] since these states can act as protective mechanisms, temporarily diverting fluorophores away from highly reactive singlet states, thereby reducing photobleaching and improving photostability to a great extent [11]. The impact of ISC becomes more pronounced and can be controlled through incorporation of heavy atoms, such as platinum, gold, bromine or iodide, into the molecular structure of fluorophores [12, 13]. This physical phenomenon is known as internal heavy atom effect taking place due to spin‐orbit coupling, e.g. a relativistic interaction between magnetic field and magnetic moment of electron's spin [14]. Spin‐orbit coupling facilitates the ISC process for heavy atom‐containing fluorophores by allowing them to perform a transition from the excited singlet state to the excited triplet state more rapidly [15]. Additionally, spin‐orbit coupling can be promoted externally upon the cascading addition of heavy ions into solution instead of decorating the molecular structure of dye by heavy atoms [16]. Understanding both internal and external heavy atom effects on ISC is crucial in the field of fluorescence spectroscopy and can provide further insights into the behavior of complex molecular systems as well as the design of new fluorescent dyes with desirable properties. Up to date, single molecule detection (SMD) methods have been extensively employed to investigate the photo‐physical transitions of most organic dyes [17, 18]. SMD necessitates fluorescent molecules with remarkably high brightness to perform a robust analysis, however, fluorophores with high triplet quantum yield have lower fluorescence brightness that pose a major limitation for SMD. In addition to SMD, fluorescence correlation spectroscopy (FCS) which detects fluorescence intensity fluctuations of relatively higher numbers of fluorescent emitters can be used to reveal dark states of fluorophores including singlet–triplet and other long‐lived transient states [19].

FCS measurements, as reliant on SMD conditions, are fundamentally tied to the molecular brightness of fluorophores [20]. When fluorescent molecules possess a higher triplet quantum yield, their fluorescence brightness will be weaker, making the analysis of their singlet‐triplet transitions more intricate. Even though intensifying the excitation power of laser can boost the brightness of fluorophores, this enhancement is curbed by fluorescence saturation originating from the build‐up of triplet states [21]. As the time frame for this build‐up shortens, it may coincide with the anti‐bunching relaxation of the fluorophores, further undermining the signal‐to‐noise conditions in recorded FCS curves. Additionally, contribution of fluorescent species to an FCS curve is directly linked to the square of their molecular brightness [22]. The uneven distribution of excitation intensity within the detection volume employed in FCS experiments leads to variable contributions from different sections of the volume, which strongly influence dark state build‐ups or fluorescence saturation [23]. Consequently, the analysis of fluorophores exhibiting high triplet quantum yields becomes significantly more complex, posing limitations in deciphering increased ISC rates, evident in both SMD methods and FCS technique [24]. Since current measurement methods have certain drawbacks mainly stemming from inadequate photon statistics, saturation and inconsistent distribution of excitation beam, there is a need for a new technique that aims to improve accuracy in detection and resolve dark state transitions taking place in longer time scales between μs and ms. To bridge this gap and tackle with aforementioned issues, in the present research, we propose an idea on a novel microfluidics‐based widefield fluorescence microscope working with a single top flat excitation beam that can capture dark transient states due to heavy atoms. This technique was recently devised and built on an optical setup with an aim to study trans‐cis isomerization kinetics of cyanine‐5 dyes and dye‐tagged vesicles, having only a single dark cis state [25]. Relying on the working principles of this state‐of‐the‐art technique, we propose a new optical setup having different optical parameters, which can detect more than one dark transient state forming at different timescales. Detection capability and sensitivity of the proposed method in capturing dark transient state build‐ups are demonstrated through numerical simulations considering electronic state model of the studied fluorophores with photophysical transitions rates.

Our suggested method is very straightforward, and the detection principle relies on tracking changes in normalized fluorescence signals of fluorescent entities constantly flowing over a uniform, top flat widefield excitation laser with supergaussian beam profile where these changes can be recorded by a sCMOS camera during laminar flow of molecules. Upon a photoexcitation over a top flat beam, flowing fluorophores will experience almost identical excitation irradiances, driving them to undergo their dark transient states that can manifest as broadening, shift or decay in recorded signals. Resultant data will then be collected as a large stack of images that contain fluorescence intensities being stored as numbers in each pixel of the images. Proposed method will provide two useful advantages; on the one hand, time‐averaged fluorescence signals, which display how dark transient states evolve under different conditions, will be retrieved from high numbers of image frames recorded during constant flow, resulting in sufficient photon statistics. On the other hand, the risk of photobleaching will be significantly reduced since each fluorescent molecule will pass over excitation beam area only once, leading to an accurate quantitative analysis. To demonstrate the concept, numerical solutions of electronic state model proposed for carboxyfluorescein (CFl) and its brominated derivatives with high triplet quantum yields which are mono‐bromo‐carboxyfluorescein (CFl‐1Br), dibromo‐carboxyfluorescein (CFl‐2Br), and tetrabromo‐carboxyfluorescein (CFl‐4Br), also known as eosin‐Y, molecules [26] are used to simulate the normalized fluorescence signals in microfluidics channel. As the simulations of signals are adapted to a widefield fluorescence microscopy integrated with a microfluidics system, geometrical dimensions of microfluidics channel, excitation profile of laser, spatial dimensions of camera images and optical parameters of widefield microscope are considered as complementary simulation parameters. Relative changes in simulated fluorescence signals in response to varying excitation laser irradiances and flow rates evidenced that dark transient states including triplet and redox (radical) states of CFl and its brominated derivatives can be resolved clearly. Preliminary computational findings presented in our study therefore suggest that similar numerical simulations can be further adapted to other fluorescent molecules with known transition rates, enabling researchers to predict whether the studied molecules with high triplet quantum yield can be displayed before performing an experiment on‐site. Our method also provides a basis and has great potential for detection of long‐lived electronic transitions of PDT agents extensively used in biomedical research. Details of photophysical model proposed for CFl, CFl‐1Br, CFl‐2Br, and CFl‐4Br molecules, and how presented method is combined with numerical simulations were outlined in the following sections.

## 2 Photophysical Model With Electronic Transition Rates

**FIGURE 1:**
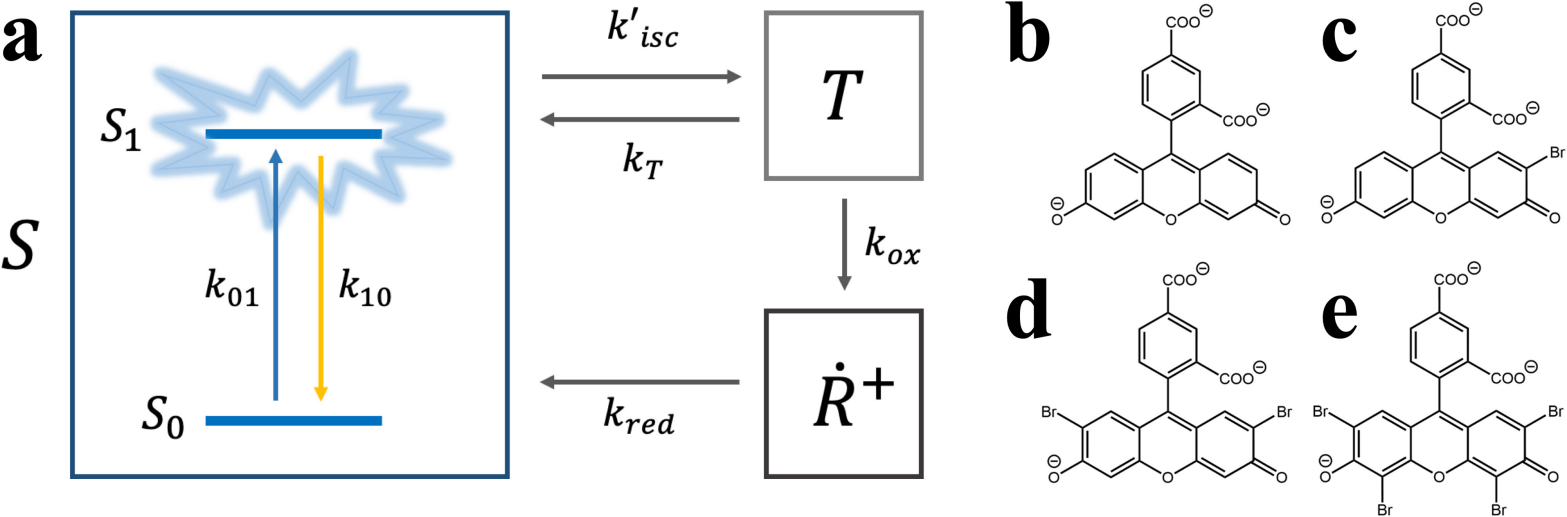
**(a)** A simplified electronic state model comprising S, T and ${\dot{R}}^{+}\left(t\right)$ states proposed for studied fluorescent probes where emission of photons takes place from emissive $S_{1}$ state to $S_{0}$ state with a de‐excitation rate, $k_{10}$. Molecular structures of **(b)** CFl, **(c)** CFl‐1Br, **(d)** CFl‐2Br, and **(e)** CFl‐4Br (eosin‐Y), respectively.

Prior to simulate the fluorescence intensity signal for fluorescent dyes flowing in microfluidic platform, one first needs to establish a photophysical (electronic state) model with transition rates for studied molecules. We used 3‐electronic state model as presented in Figure 1a, that was previously developed for both CFl and its brominated derivatives in ref. [27] whose molecular structures were presented in Figure 1b–e. In the model, a singlet state S which has a ground singlet state ($S_{0}$) and an emissive first singlet excited state ($S_{1}$), a triplet (dark) state (T) and a photooxidized (dark) radical state (${\dot{R}}^{+}\left(t\right)$) are involved. As relatively lower excitation irradiances are applied in proposed widefield microscopy operating with wider excitation beam profile, higher excited singlet and triplet states were disregarded from the model. In accordance with the given model, in S state, fluorophores residing in $S_{0}$ are first excited to $S_{1}$ with $k_{01}$ rate given by Equation 1 as below:

(1) $$k_{01}=\sigma_{\mathrm{exc}}\cdot \Phi_{\mathrm{exc}}=\sigma_{\mathrm{exc}}\cdot \frac{I_{\mathrm{exc}}}{E}$$

where the terms $\sigma_{\mathrm{exc}}$, $\Phi_{\mathrm{exc}}$, $I_{\mathrm{exc}}$ and E account for excitation cross section of the dye, local excitation flux, excitation irradiance of laser beam and energy of incident photons, respectively [14]. Radiative decay, e.g. fluorescence emission, from $S_{1}$ to $S_{0}$ is presented by the de‐excitation rate, $k_{10}$. ISC takes place from a singlet S state to T by effective $k_{\mathrm{isc}}^{\prime }$ rate that can be written as follows:

(2) $$k_{\mathrm{isc}}^{\prime }=k_{\mathrm{isc}}\cdot \frac{k_{01}\left(t\right)}{k_{01}\left(t\right)+k_{10}}$$

where $k_{\mathrm{isc}}$ is an intrinsic ISC rate. Moreover, $k_{T}$ is the triplet relaxation rate from T to $S_{0}$. Photooxidation of fluorophores to ${\dot{R}}^{+}\left(t\right)$ state is presumed to emerge from T with $k_{\mathrm{ox}}$ rate. From ${\dot{R}}^{+}\left(t\right)$ state which has much longer lifetime than $S_{1}$, the fluorescent molecules relax back to $S_{0}$ via a photoreduction rate, $k_{\mathrm{red}}$. Using the model, de‐excitation rate can be calculated by Equation 3 as the following:

(3) $$k_{10}=1/\tau_{f}-k_{\mathrm{isc}}^{\prime }-k_{\mathrm{ox}}$$

where $\tau_{f}$ is a fluorescence lifetime of the fluorophore. Since $S_{1}$ state is the only emissive state in the presented photophysical model, time‐dependent $S_{1}$(*t*) population was computed in the numerical simulations of fluorescence signals.

## 3 Computational Methods and Analyses

**FIGURE 2:**
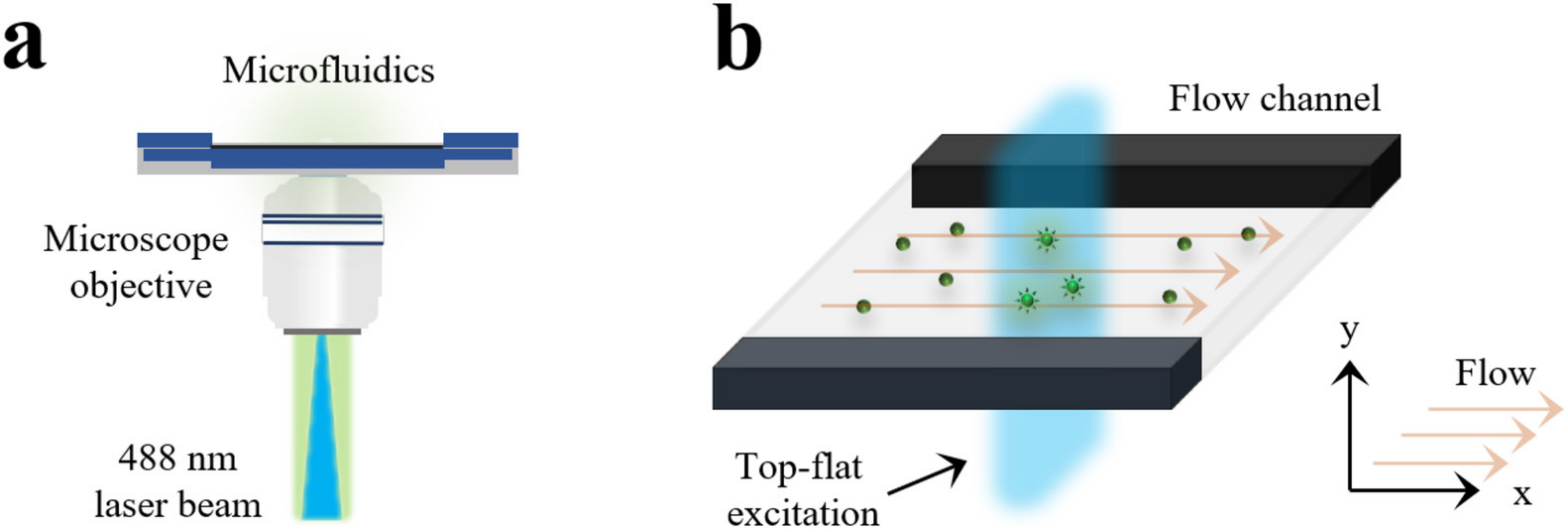
Schematic illustration of microfluidics platform placed on a microscope objective of a proposed widefield fluorescence microscope. **(a)** Fluorophores are excited via a stationary, continuous 488 nm excitation beam (colored blue) focused at the back aperture of microscope objective. The resulting collimated fluorescence emission (colored light‐green) is collected by sCMOS camera. **(b)** The position of a flow channel embedded within the microfluidics cage. Top flat excitation beam (colored blue) stretched across the flow direction will excite all fluorescent molecules flowing over a stationary laser beam and drive all fluorophores to undergo their long‐lived T and ${\dot{R}}^{+}$ states. Arrows show the direction of laminar flow exerted on molecules along x‐axis.

In a proposed experimental method, a microfluidic platform is aimed to be combined with a widefield fluorescence microscope, as visualized in Figure 2a, where fluorescence signal is continuously recorded from the flowing fluorophores. The snapshots of the fluorophores flowing over a uniform, stationary excitation beam can be recorded by a sCMOS camera [28] and fluorescence intensity image $\left\langle F\left(x,y\right)\right\rangle$ can be produced from a large stack of frames (preferably more than 500 frames to maintain sufficient statistics) where x and y denote the spatial dimensions along flow direction and across the flow channel, respectively. With information of flow rate and size of square pixels in a recorded image, one can compute the time it takes for an ensemble of fluorescent molecules to pass one single pixel. The fluorescence signal along flow direction is denoted by $\left\langle F\left(x\right)\right\rangle$ and can be converted to time‐averaged fluorescence signal $\left\langle F\left(t\right)\right\rangle$ using a fundamental equation t=x/v where v is the ratio of flow rate to cross‐section area of a microfluidic cell. By widefield imaging the microfluidic channel that resides on a microscope objective, the time averaged fluorescence signal, $\left\langle F\left(t\right)\right\rangle$ will be detected from an ensemble of fluorescent molecules flowing with a constant laminar flow rates ranging between 100 μL/min and 2000 μL/min. Based on applied flow rates, geometry of microfluidic channel and local excitation conditions, relative changes in normalized time‐averaged fluorescence intensity signal are expected to be observed when the fluorophores undergo long‐lived, dark transient states. To properly detect $\left\langle F\left(t\right)\right\rangle$ signal of fluorophore originating from the emissive $S_{1}$ state, which can be directly populated by $k_{01}$, one must consider the geometry of a flow cell as well as the excitation profile of the laser beam. In the present work, excitation beam profile together with normalized fluorescence signals along flow direction are computed as image data for each studied fluorophore under different environmental conditions and simulations were performed in Jupyter Anaconda, Python environment [29]. Theoretical considerations and computational details were described in Sections 3.1 and 3.2.

### 3.1 Selection of Experimental Parameters and Simulation of a Top Flat Excitation Beam in a Flow Cell

The main hypothesis of proposed experimental framework is that all flowing fluorescent entities are assumed to pass over a uniform excitation beam where the spatial distribution of excitation irradiance is very similar across the flow direction as illustrated schematically in Figure 2b. Excitation wavelength ($\lambda_{\mathrm{exc}}$) of a continuous laser source is selected as 488 nm since CFl and its brominated derivatives can absorb excitation light to a great extent at this specific wavelength [27]. Considering acquisition settings and standard field of view (FOV) dimensions of a typical sCMOS camera, length and width of flow channel can be set to 221.86 μm, whereas the height in flow channel is selected as 50 μm. Images which aimed to be recorded via sCMOS camera consist of 2048 square pixels in both horizontal and vertical dimensions where each pixel is 6.5 μm in size [30], however, the total number of pixels in each dimension is set to 512 via 4×4 binning in order to increase the signal‐to‐noise ratio and the measurement speed when recording the frames from flowing fluorophores [31]. The pixel size on sample plane where molecules move with a constant flow rate can be translated to μm from pixels when binned pixel size (26 μm) is divided by the magnification factor of the microscope objective. As a water immersion objective with 60x magnification installed on microscope is considered in design of proposed experimental setup, the size of each pixel is calculated to be 0.43 μm/pixel. Therefore, horizontal and vertical dimensions of image data are calculated to be 221.86 μm long. In python environment, 2D grid is created to simulate the excitation beam at the center coordinates of simulated image considering these dimensions [32]. A flattop beam profile can be produced by a supergaussian profile [33] and the optical intensity profile of the beam order n can be expressed by Equation 4 as follows:

(4) $$I\left(r\right)=I_{0}\cdot e^{-2{\left(r/w\right)}^{n}}$$

where r represents the spatial dimension (also referring to a combination of x and y in 2D cartesian coordinate system), $I_{0}$ is the magnitude of optical intensity and w is the beam waist radius in both *x* and *y* axes [34, 35].

**FIGURE 3:**
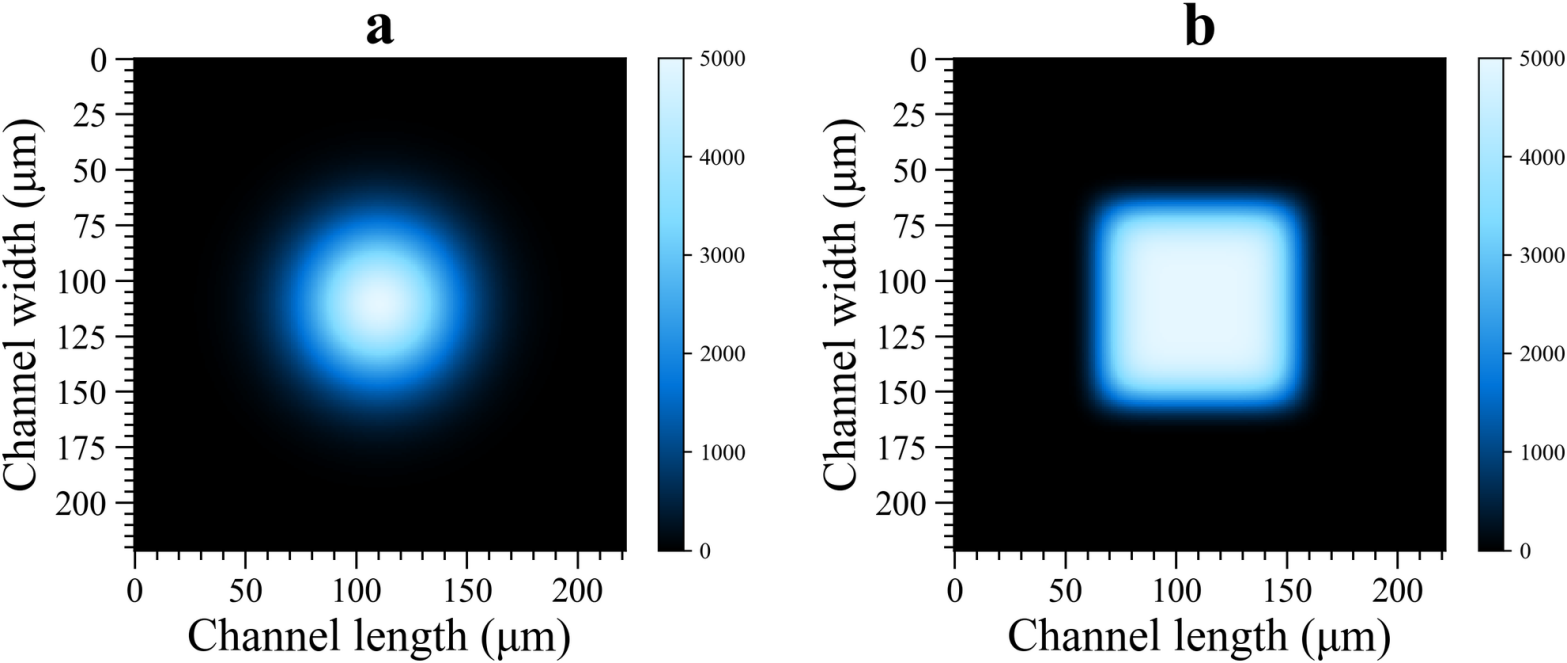
Simulated optical intensity profile images of **(a)** a well‐round, uniform Gaussian beam profile (n= 2) and **(b)** a supergaussian, top flat beam profile (n = 6) with a squared area geometry.

**FIGURE 4:**
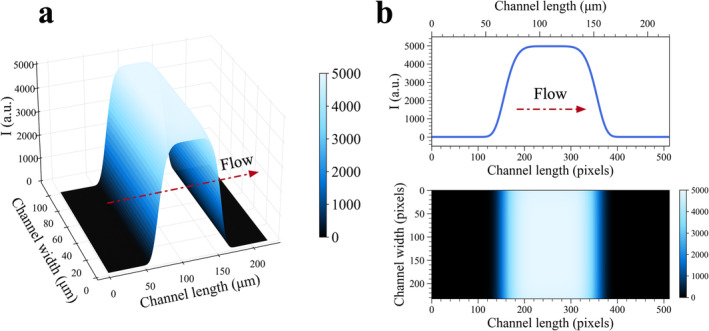
Numerical simulations of a supergaussian top flat beam profile stretched across flow direction (L =100 μm) in microfluidic channel. **(a)** 3D image of a simulated excitation beam and **(b)** a top‐view of excitation beam area in flow cell where mean value of I signal along flow direction is plotted above heatmap. Colorbars on the right represent the evolution of I at different positions along and across flow direction.

For a well‐round, uniform Gaussian beam profile, n takes the value of 2 whereas n > 2 is considered for flattop beam profiles [36]. The greater the n values, the sharper the edges of a flattop beam profile with a squared area geometry. A stationary excitation beam with Gaussian profile can be engineered into top flat beam profiles by using Powell lens pairs combined with cylindrical lenses in optical setups [37]. Images of a perfectly round Gaussian beam (n = 2) and a strong flattop beam (n= 6) profile, each having w = 50 μm and $I_{0}$ = 5000 a.u., were simulated in the FOV by using Equation 4 and produced images were presented in Figure 3. In design of a flow cell used in computational experiments, a flow channel with the same channel length but a narrower channel width (L = 100 μm) was considered, and top flat beam profile simulated in Figure 3b was stretched across the flow direction in this design. Thereby, all fluorescent molecules passing over top flat beam will experience photoexcitation and dark transient states will be populated. Further extension of beam dimensions leads to a reduction in $k_{01}$ rate, resulting in inadequate excitation of fluorophores. Therefore, the channel width of FOV simulated in Figure 3b was truncated from L = 221.86 to L = 100 μm. As an excitation beam, a top flat Gaussian beam (n = 6), which has $I_{0}$ = 5000 a.u. with a beam waist radius, w = 50 μm and vertical beam radius stretched across the flow direction, L = 100 μm was employed in all computational experiments. Detailed discussions regarding the influence of beam dimensions on simulated fluorescence images can be found from Section 4. A general form of $I\left(x,y\right)$ that yields such a stretched beam profile can then be expressed by Equation 5 as given below and simulated beam together with its mean intensity signal computed with respect to flow direction was visualized in Figure 4.

(5) $$I\left(x,y\right)=\left\{\begin{array}{c} I_{0}\cdot e^{-2{\left(\frac{x-x_{0}}{w}\right)}^{6}}\;\;y\;\epsilon \;\left[0,L\right]\\ \begin{array}{cc} \;0 & \;\text{otherwise} \end{array} \end{array}\right.$$

where the peak optical intensity of the simulated beam is $I_{0}/e^{2}$ and $x_{0}$ denotes the center coordinate of the beam along channel length. Effective optical power, $P_{\mathrm{eff}}$ of the laser beam applied to excite the fluorescent molecules on the sample plane has a strong dependence on $I\left(x,y\right)$ and $P_{\mathrm{eff}}$ can be calculated by the integral of $I\left(x,y\right)$ over the area of the laser beam as the following:

(6) $$P_{\mathrm{eff}}=\int_{-\infty }^{\infty }\int_{0}^{L}I_{0}e^{-2{\left(\frac{x-x_{0}}{w}\right)}^{6}}\;\mathrm{dy}\;\mathrm{dx}$$

Explicit solution of Equation 6 will then produce $I_{0}$ in terms of $P_{\mathrm{eff}}$ together with spatial dimensions of the beam, w and L, as given below:

(7) $$I_{0}=\frac{3\sqrt[{6}]{2}}{\Gamma \left(1/6\right)}\frac{P_{\mathrm{eff}}}{\mathrm{wL}}$$

where the gamma function is denoted by Γ and $\Gamma \left(1/6\right)$ can be approximated to a numerical value of ≃ 5.57 [38]. For numerical simulations of fluorescence signals under different excitation conditions, $I_{0}$ will be regarded as a peak value of excitation irradiance which will directly alter $k_{01}$, therefore, the dark‐state populations of fluorophores studied in the present research. As continuous wavelength (CW) diode lasers operating at $\lambda_{\mathrm{exc}}$ = 488 nm can produce maximum output power up to 300 mW, $P_{\mathrm{eff}}$ values tested in computational experiments were varied in the range between 5 mW and 300 mW. $I_{0}$ values computed for top flat beam profiles of different w values in this $P_{\mathrm{eff}}$ range and further information related to computational data can be found from supplementary file (Section 1 and Tables S1 and S2).

### 3.2 Computation of a Time‐Averaged Fluorescence Intensity Signal Along Flow Direction Using Analytical Solution of a Photophysical Model

Having established the electronic state model for fluorophores and excitation beam geometry in a microfluidic channel, one can simulate fluorescence signal for specimens through computing S state (containing both $S_{0}$ state and emissive $S_{1}$ state) in terms of photophysical transition rates. Electronic state model presented in Figure 1a can be treated as a system of first order ordinary differential equations which can be expressed in a generalized form as the following:

(8) $$\frac{d}{\mathrm{dt}}\bar{P}\left(t\right)=M\cdot \bar{P}\left(t\right)$$

where $\bar{P}\left(t\right)$ and M are regarded as a population vector of electronic states and a coupling matrix comprising photophysical transition rates between different electronic states, such that:

(9) $$\frac{d}{\mathrm{dt}}\left[\begin{array}{c} S\left(t\right)\\ T\left(t\right)\\ {\dot{R}}^{+}\left(t\right) \end{array}\right]=\left[\begin{array}{ccc} -k_{\mathrm{isc}}^{\prime } & k_{T} & k_{\mathrm{red}}\\ k_{\mathrm{isc}}^{\prime } & -k_{\mathrm{ox}}-k_{T} & 0\\ 0 & k_{\mathrm{ox}} & -k_{\mathrm{red}} \end{array}\right]\cdot \left[\begin{array}{c} S\left(t\right)\\ T\left(t\right)\\ {\dot{R}}^{+}\left(t\right) \end{array}\right]$$

Upon treating the electronic state populations as probabilities, one must satisfy the condition $S\left(t\right)+T\left(t\right)+{\dot{R}}^{+}\left(t\right)=1\;\forall t$≥ 0. Since the fluorophores are not yet photoexcited by the laser beam at t = 0 s and thereby reside completely in *S* state, the initial condition for electronic state vector can be set as:

(10) $$\bar{P}\left(t=0\right)=\left[\begin{array}{c} S\left(t=0\right)\\ T\left(t=0\right)\\ {\dot{R}}^{+}\left(t=0\right) \end{array}\right]=\left[\begin{array}{c} 1\\ 0\\ 0 \end{array}\right]$$

Under a constant excitation irradiance initiated right after t=0 s, the probability for a fluorescent molecule to be in either $S_{0}$ or $S_{1}$ state of S at any time t can be expressed by Equation 11:

(11) $$S\left(t\right)=1-\sum_{n=1}^{p}\left(A_{n}-A_{n}e^{\lambda_{n}t}\right)$$

where $\lambda_{n}$ is the eigenvalue, also referring to relaxation mode rate of $\bar{P}\left(t\right)$ upon onset of constant photoexcitation, p is the number of different dark transient states, $A_{n}$ is the related amplitude which represents the population of different nonfluorescent states at steady state ($t\gg 1/\lambda_{n}$). Upon photoexcitation by a laser beam, equilibration between the $S_{1}$ and $S_{0}$ states occurs within the fluorescence lifetime (on the nanosecond scale), governed by the so‐called antibunching relaxation time. This relaxation process is usually averaged out over the longer time scales of long‐lived, nonfluorescent state transitions observed over TRAST microscopy, and this is also the case in our suggested method. Using an initial condition given by Equation 10, both $\lambda_{n}$ and $A_{n}$ can be solved, analytically for the S (comprising both $S_{0}$ and $S_{1}$) state as well as the nonfluorescent T and ${\dot{R}}^{+}\left(t\right)$ states. Characteristic relaxations between T and $S_{0}$ states have typical timescales of μs while relaxations from nonfluorescent ${\dot{R}}^{+}\left(t\right)$ state to $S_{0}$ state take place between μs and ms. Having solved the eigenvalues and eigenvectors of M by using Equations 9 and 10, one can calculate the singlet state population S by Equation 12 as follows:

(12) $$S\left(t\right)=A_{1}e^{\lambda_{1}t}+A_{2}e^{\lambda_{2}t}+A_{3}e^{\lambda_{3}t}$$

where the amplitudes $A_{1}$, $A_{2}$ and $A_{3}$ can be written in terms of transition rates as the following:

(13) $$A_{1}=\frac{k_{T}k_{\mathrm{red}}}{k_{\mathrm{isc}}^{\prime }\left(k_{\mathrm{ox}}+k_{\mathrm{red}}\right)+k_{\mathrm{red}}k_{T}}$$

(14) $$A_{2}=\frac{k_{\mathrm{isc}}^{\prime }}{k_{\mathrm{isc}}^{\prime }+k_{T}}$$

(15) $$A_{3}=\frac{k_{\mathrm{isc}}^{\prime }k_{T}k_{\mathrm{ox}}}{\left(k_{\mathrm{isc}}^{\prime }\left(k_{\mathrm{ox}}+k_{\mathrm{red}}\right)+k_{\mathrm{red}}k_{T}\right)\left(k_{T}+k_{\mathrm{isc}}^{\prime }\right)}$$

The eigenvalues $\lambda_{1}$, $\lambda_{2}$ and $\lambda_{3}$ are obtained as listed below:

(16) $$\left[\begin{array}{c} \lambda_{1}\\ \lambda_{2}\\ \lambda_{3} \end{array}\right]=\left[\begin{array}{c} 0\\ -k_{\mathrm{isc}}^{\prime }-k_{T}\\ -k_{\mathrm{red}}-k_{\mathrm{ox}}\frac{k_{\mathrm{isc}}^{\prime }}{k_{\mathrm{isc}}^{\prime }+k_{T}} \end{array}\right]$$

Experimentally, the time‐averaged fluorescence intensity $\left\langle F\left(t\right)\right\rangle$ signal detected from the flowing fluorophores via sCMOS camera under a stationary excitation with top flat beam profile can be approximated by:

(17) $$\left\langle F\left(t\right)\right\rangle =q_{D}q_{F}k_{10}S_{1}\left(t\right)=k_{10}q_{D}q_{F}S\left(t\right)\frac{k_{01}\left(t\right)}{k_{01}\left(t\right)+k_{10}\left(t\right)}$$

where the parameters $q_{D}$ and $q_{F}$ represents the quantum detection efficiency of the camera and the fluorescence quantum yield of the fluorophores, respectively [25]. However, when simulating a fluorescence signal in the present work, normalization of $\left\langle F\left(t\right)\right\rangle$ with respect to its peak value ${\left\langle F\right\rangle }_{\mathrm{norm}}=\left\langle F\left(t\right)\right\rangle /{\left\langle F\left(t\right)\right\rangle }_{\max}$ will exterminate the contributions originating from $q_{D}$ and $q_{F}$. The relative changes in simulated ${\left\langle F\right\rangle }_{\mathrm{norm}}$ curves will decipher the population dynamics of long‐lived, nonfluorescent states under different environmental conditions. Broadening, shifts and decays seen in ${\left\langle F\right\rangle }_{\mathrm{norm}}$ signals simulated for fluorophores can thereby be treated as a dark state indicator when compared to excitation profile of laser beam which has electronic transitions only between singlet $S_{0}$ and $S_{1}$ states. Electronic transition rates, laminar flow rates together with corresponding passage times, and $I_{0}$ values used in simulations of ${\left\langle F\right\rangle }_{\mathrm{norm}}$ signals were detailed in Section 4.

## 4 Simulation Results and Discussions

Population dynamics of dark transient states of fluorescent molecules are strongly dependent on excitation powers applied to molecules in detection volume created over microscope objectives [21]. It is very important that the overall impact of the excitation light intensity and its duration on dark transient states can vary depending on the photophysical characteristics of the fluorophore as well as the experimental conditions. Experimental design and rigorous monitoring of the fluorophore's behavior under different illumination conditions are essential in revealing dark state dynamics and can be optimized well by taking advantage of photophysical transitions of studied molecules, e.g. electronic transition rates of fluorophores. For the numerical simulations of fluorescence signals in our proposed microfludics‐based monitoring technique, fluorescence lifetime data, excitation cross‐sections and electronic transition rates including $k_{T}$, $k_{\mathrm{isc}}$, $k_{\mathrm{red}}$ and $k_{\mathrm{ox}}$ were collected for CFl, CFl‐1Br, CFl‐2Br and CFl‐4Br from one of our recent research [27], and these values were presented in Table 1. Since tabulated values were acquired globally from power series experiments, these rates were fixed in the numerical simulations of ${\left\langle F\right\rangle }_{\mathrm{norm}}$ signals while $I_{0}$ and flow rates were varied.

**TABLE 1:** Fluorescence lifetime (*τ*_f_) data, excitation cross‐sections (*σ*_*exc*_) and electronic transition rates that were experimentally acquired for CFI, CFl‐1Br, CFl‐2Br, and CFl‐4Br by TRAST spectroscopy [27].

Fluorophore	Lifetime (ns)	$\sigma_{\mathrm{exc}}$ (*cm* ^2^)	$k_{\mathrm{isc}}$ (${\mu s}^{-1}$)	$k_{T}$ (${\mu s}^{-1}$)	$k_{\mathrm{red}}$ (${\mu s}^{-1}$)	$k_{\mathrm{ox}}$ (${\mu s}^{-1}$)
CFl	4.04	$3\cdot 10^{-16}$	10.8	0.71	0.00001	0.00124
CFl‐1Br	2.71	$2.52\cdot 10^{-16}$	94.5	0.61	0.0000104	0.000433
CFl‐2Br	1.77	1.77$\cdot 10^{-16}$	309	0.61	0.00047	0.000238
CFl‐4Br*(Eosin‐Y)	1.12	$1.65\cdot 10^{-16}$	893	0.58	0.005	0.00001

* CFl‐4Br is also known as eosin‐Y.

**FIGURE 5:**
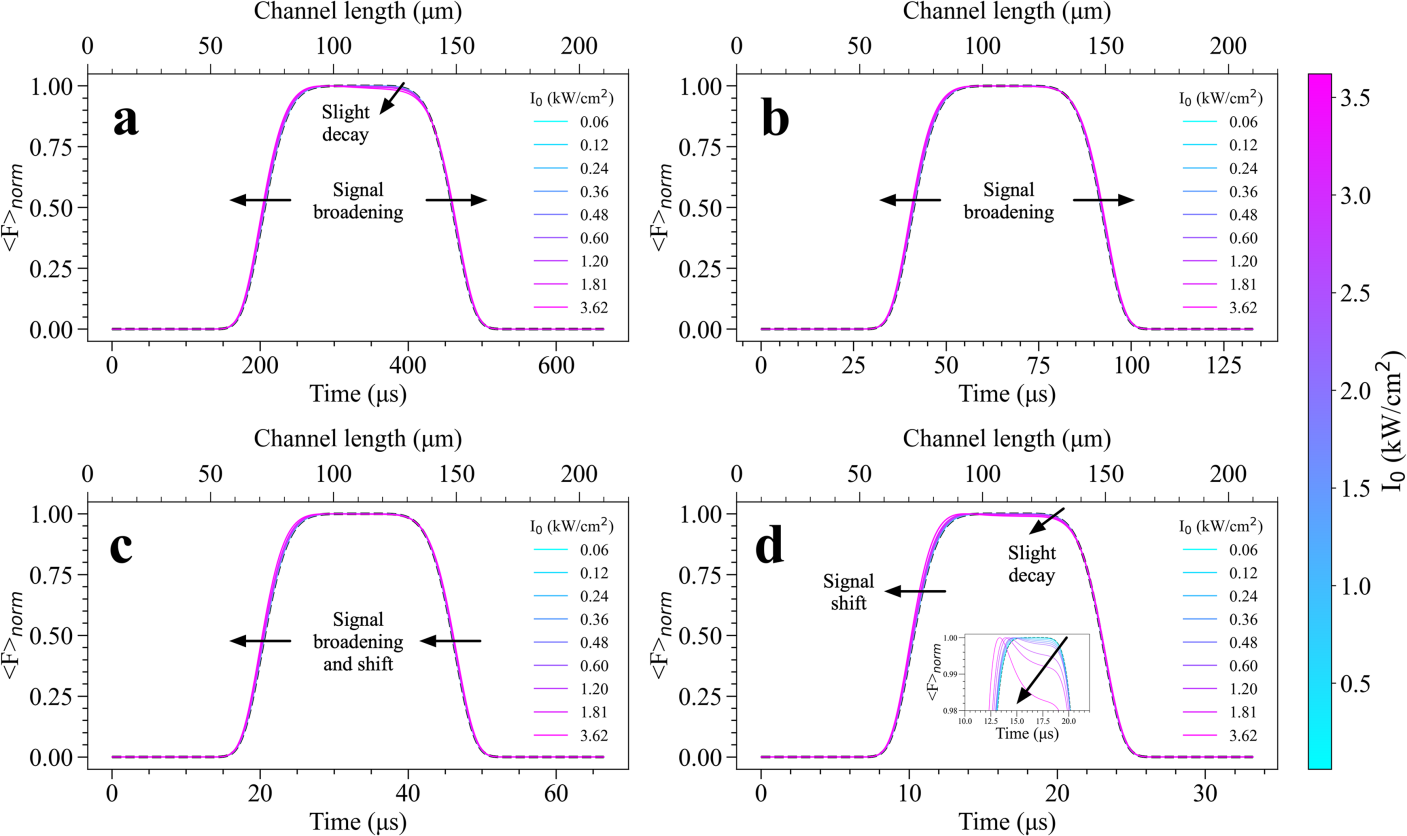
The influence of excitation irradiance, $I_{0}$ varying from 0.06 to 3.62 kW/cm^2^ on ${\left\langle F\right\rangle }_{\mathrm{norm}}$ signals of bromine‐free CFl molecules flowing under constant flow rates of **(a)** 100, **(b)** 500, **(c)** 1000, and **(d)** 2000 μL/min, respectively. In figures, passage times of fluorophores over excitation beam computed for different flow rates were given in lower x‐axis, colorbar shows $I_{0}$ values and dashed black line shows the normalized beam profile signal (that has no dark state build‐up) as presented in Figure 4b.

To demonstrate the working principle of proposed methodology and show how ${\left\langle F\right\rangle }_{\mathrm{norm}}$ signals alter in response to different $I_{0}$ values at varying laminar flow speeds, numerical simulations were first carried out for bromine‐free CFl molecules that has longer fluorescence lifetime than its brominated variants. Simulated ${\left\langle F\right\rangle }_{\mathrm{norm}}$ curves at different $I_{0}$ values in the range between 0.06 and 3.62 kW/cm^2^ were presented in Figure 5 for four different flow rates applied, which are (a) 100, (b) 500, (c) 1000, and (d) 2000 μL/min, respectively. For the lowest flow rate, that is 100 μL/min, the increase in $I_{0}$ from 0.06 to 3.62 kW/cm^2^ leads to a slight broadening of ${\left\langle F\right\rangle }_{\mathrm{norm}}$ signals of CFl outward the laser beam profile (shown by a dashed black line in Figure 5a). This slight fluorescence broadening arouse in simulated signals can serve as an indicator for the presence of dark transient states. A slight decay in ${\left\langle F\right\rangle }_{\mathrm{norm}}$ signals was additionally seen in the direction of laminar flow. These signal broadening and decay behaviors can be attributed to the build‐up of both long‐lived, dark triplet and photooxidation states, as were experimentally observed via stationary TRAST spectroscopy [27]. The passage times of CFl molecules which pass over a uniform, top flat excitation beam at 100 μL/min give them sufficient time to undergo ${\dot{R}}^{+}\left(t\right)$ state since transitions to ${\dot{R}}^{+}\left(t\right)$ state is slower compared to T state which occurs in only a few microseconds. For that reason, broadening and decays seen in ${\left\langle F\right\rangle }_{\mathrm{norm}}$ signals mostly reflect ${\dot{R}}^{+}\left(t\right)$ state build‐up at 100 μL/min.

To further investigate whether dark T and longer‐lived ${\dot{R}}^{+}\left(t\right)$ states can be resolved more concisely, flow rate in microfluidics platform was increased to 500 μL/min and ${\left\langle F\right\rangle }_{\mathrm{norm}}$ signals were simulated using the same $I_{0}$ values. As can be clearly seen in Figure 5b, the passage time of fluorophores which move in the direction of laminar flow becomes approximately five times shorter when compared to flow rate of 100 μL/min, thus, fluorescent molecules passing the excitation beam area are much faster. As a result, there is a prominent recovery shown up in ${\left\langle F\right\rangle }_{\mathrm{norm}}$ signals of CFl and no further decay was seen in ${\left\langle F\right\rangle }_{\mathrm{norm}}$ signals upon an increase in $I_{0}$ values from 0.06 to 3.62 kW/cm^2^. As the passage times of fluorophores get faster at higher flow rates, the population of ${\dot{R}}^{+}\left(t\right)$ state is expected to be much lower compared to 100 μL/min, resulting in a recovery in ${\left\langle F\right\rangle }_{\mathrm{norm}}$ signal. However, the broadening of ${\left\langle F\right\rangle }_{\mathrm{norm}}$ signals at higher $I_{0}$ under 500 μL/min can still be associated with the presence of dark‐transient states. At relatively higher flow speed of 1000 μL/min as presented in Figure 5c, not only broadening of signals, however, also a signal shift in simulated ${\left\langle F\right\rangle }_{\mathrm{norm}}$ signals could be observed upon an increment in $I_{0}$. This is another indicator demonstrating the presence of dark transient state populations and these shifts present in ${\left\langle F\right\rangle }_{\mathrm{norm}}$ signals become more distinguishable at higher flow rates such as 2000 μL/min (see Figure 5d). In our proposed microfluidics‐based dark transient state detection method, within much shorter time scales which can be achieved by applying high flow rates including 1000 μL/min and 2000 μL/min, some fraction of fluorophores still resides in their dark T and ${\dot{R}}^{+}\left(t\right)$ states with longer lifetime, therefore, the relative changes in ${\left\langle F\right\rangle }_{\mathrm{norm}}$ signal could be resolved and in turn dark state build‐ups became traceable when higher excitation powers were applied. As shown in Figure 5d, non‐complete recovery of CFl molecules to their singlet $S_{0}$ state became visible through slight decays in ${\left\langle F\right\rangle }_{\mathrm{norm}}$ signals and higher dark state population could be monitored at 2000 μL/min. Magnified image of the same ${\left\langle F\right\rangle }_{\mathrm{norm}}$ signals presented in the inset of Figure 5d shows the signal decays more clearly and decays become more pronounced at higher $I_{0}$ values. Signal decays shown at 2000 μL/min flow rate can be attributed to the build‐up of T state since the transitions from $S_{1}$ state to T state occur within only a few microseconds. This suggests that triplet formation can be resolved via our proposed method at this particular flow rate. Under fast flow rates including 2000 μL/min, changes in ${\left\langle F\right\rangle }_{\mathrm{norm}}$ signals can become more distinguishable when the beam size is shrunk. Additional ${\left\langle F\right\rangle }_{\mathrm{norm}}$ signal simulations performed under 2000 μL/min flow rate at varying $I_{0}$ values have suggested that signal decays and shifts become stronger when smaller beam waist radius values of w = 30 μm and w = 40 μm are used in excitation beam (see Figure S1). The lower the beam size, the stronger the $I_{0}$ values can populate more dark states, leading to strong changes in ${\left\langle F\right\rangle }_{\mathrm{norm}}$ signals. These computational outcomes suggest that monitoring triplet and photooxidation build‐ups are strongly dependent on excitation powers, flow rates as well as the dimensions of excitation beam used in microfluidics channel.

**FIGURE 6:**
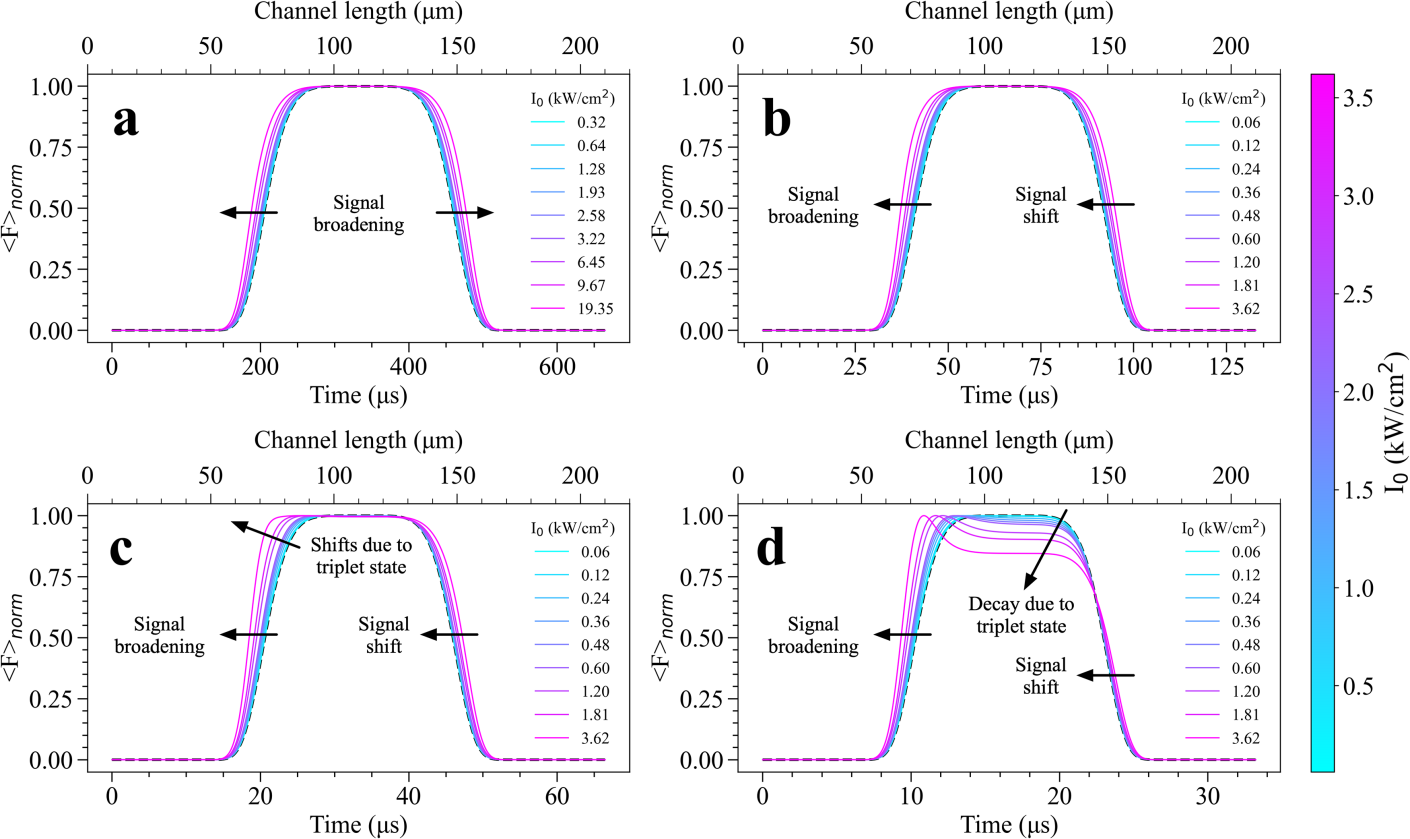
The influence of excitation irradiance, $I_{0}$ varying from 0.06 to 3.62 kW/cm^2^ on ${\left\langle F\right\rangle }_{\mathrm{norm}}$ signals of CFl‐4Br (eosin‐Y) molecules flowing under constant flow rates of **(a)** 100, **(b)** 500, **(c)** 1000, and **(d)** 2000 μL/min, respectively. In figures, passage times of fluorophores over excitation beam computed for different flow rates were given in lower x‐axis, colorbar shows $I_{0}$ values and dashed black line shows the normalized beam profile signal (that has no dark state build‐up) as presented in Figure 4b.

In order to show the trackability of fluorophore blinking in our proposed method, we then simulated ${\left\langle F\right\rangle }_{\mathrm{norm}}$ signals for CFl‐4Br (eosin‐Y) molecule, which contain four heavy bromine atoms bound to molecular structure of CFl, using its electronic transition rates tabulated in Table 1. In the simulations of ${\left\langle F\right\rangle }_{\mathrm{norm}}$ signals performed for CFl‐4Br, the same excitation irradiances and flow rates used for CFl, are considered. ${\left\langle F\right\rangle }_{\mathrm{norm}}$ signals simulated for CFl‐4Br were presented in Figure 6. At the elevated $I_{0}$ values, a very prominent symmetrical broadening effect was obtained for CFl‐4Br even at the lowest flow rate of 100 μL/min (see Figure 6a), and simulated ${\left\langle F\right\rangle }_{\mathrm{norm}}$ signals were found quite distinguishable in comparison with the curves simulated for bromine‐free CFl compounds. The underlying reason behind such a pronounced broadening behavior under the same environmental conditions can be explained by internal heavy atom effect of bromine atoms. Stronger $k_{\mathrm{isc}}$ rate of CFl‐4Br along with its high triplet quantum yield, leads to enhanced spin‐orbit coupling. This results in wider signal broadening or a stronger shift in the simulated ${\left\langle F\right\rangle }_{\mathrm{norm}}$ signals. As visualized in Figure 6b, for slightly higher flow rate of 500 μL/min, the similar broadening effect was observed together with the signal shift particularly at higher $I_{0}$ values such as 1.81 and 3.62 kW/cm^2^. In addition to broadening and signal shift, decays in the amplitudes of ${\left\langle F\right\rangle }_{\mathrm{norm}}$ signals started to show up when the flow rate was increased to 1000 μL/min as seen in Figure 6c where increased dark T state population lowers ${\left\langle F\right\rangle }_{\mathrm{norm}}$ signals at this fast time scale. This reduction in ${\left\langle F\right\rangle }_{\mathrm{norm}}$ signals became more prominent at higher $I_{0}$ values as a result of much higher T state build‐up and ~20% decay could be seen for an ensemble of CFl‐4Br when the flow rate was set to 2000 μL/min (see Figure 6d).

**FIGURE 7:**
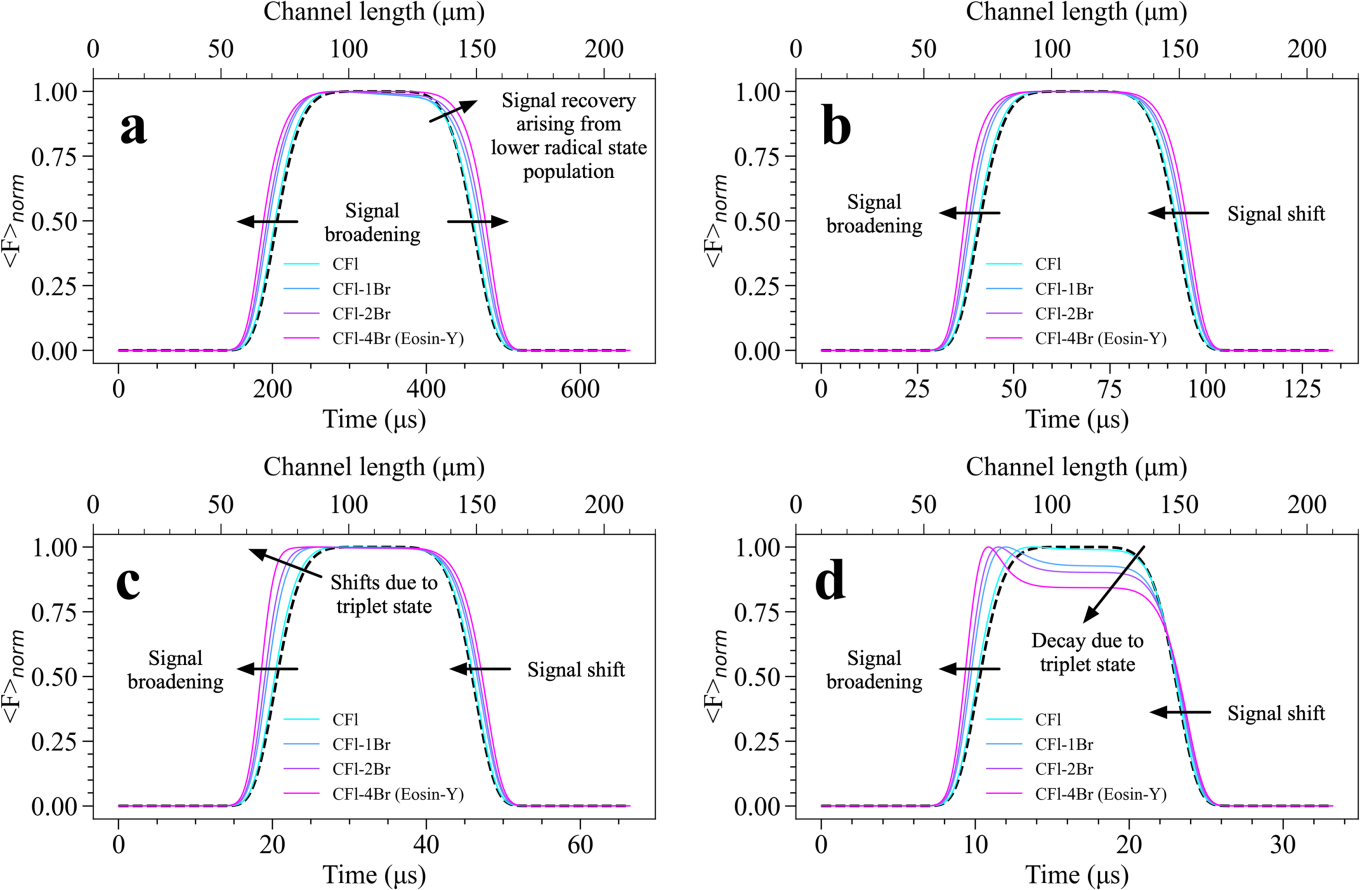
The influence of flow rate on ${\left\langle F\right\rangle }_{\mathrm{norm}}$ signals of CFl and its brominated derivatives flowing under constant flow rates of **(a)** 100, **(b)** 500, **(c)** 1000, and **(d)** 2000 μL/min, at $I_{0}$ = 3.62 kW/cm^2^. In figures, passage times of fluorophores over excitation beam computed for different flow rates were given in lower x‐axis and dashed black line in each figure shows the normalized beam profile signal (that has no dark state build‐up) as presented in Figure 4b.

Apart from the comparative assessment of bromine‐free CFl and CFl‐4Br molecules, the impact of bromination degree, defined by the number of Br atoms attached, on ${\left\langle F\right\rangle }_{\mathrm{norm}}$ signals was systematically investigated. Other CFl derivatives containing only 1 and 2 Br atoms were compared with CFl and CFl‐4Br to check whether signal broadening, shifts and decays can be promoted in ${\left\langle F\right\rangle }_{\mathrm{norm}}$ signals upon a systematic increase in the number of Br atoms attached to CFl. ${\left\langle F\right\rangle }_{\mathrm{norm}}$ signals simulated for each dye are performed at $I_{0}$ = 3.62 kW/cm^2^ under different flow rates varying between 100 and 2000 μL/min, and computational findings were summarized in Figure 7. As clearly shown in Figure 7a, ${\left\langle F\right\rangle }_{\mathrm{norm}}$ signals get broader at 100 μL/min flow rate when more Br atoms are bound to the molecular structure of CFl molecule. On the other hand, a slight decay in ${\left\langle F\right\rangle }_{\mathrm{norm}}$ signal seen for bromine‐free CFl molecule recovers when the degree of bromination increases. The effect of ${\dot{R}}^{+}\left(t\right)$ state build‐up on ${\left\langle F\right\rangle }_{\mathrm{norm}}$ signal is clearly resolved at this longer timescale, therefore, this signal recovery can be attributed to a reduction in ${\dot{R}}^{+}\left(t\right)$ state when more Br atoms are bound. This outcome is in a good agreement with TRAST experiments [27]. Under a slightly higher flow rate of 500 μL/min presented in Figure 7b, the decay effects of ${\dot{R}}^{+}\left(t\right)$ state are no longer evident due to the faster transit of molecules through the excitation beam. However, signal broadening and shifts can still be resolved for each dye separately. Under 1000 μL/min flow rate, signal broadening and spectral shifts persist across all CFl derivatives. At a flow rate of 1000 *μ*L/min (see Figure 7c), these broadening and shifting effects become more pronounced, particularly in highly brominated CFl derivatives, as the T state becomes more resolvable at shorter timescales. To resolve T state build‐up in ${\left\langle F\right\rangle }_{\mathrm{norm}}$ signals, flow rate was increased up to 2000 μL/min and signals computed at this flow rate were presented in Figure 7d. Computational results found for the highest flow rate have revealed more distinguishable decays in ${\left\langle F\right\rangle }_{\mathrm{norm}}$ signals together with further broadening and shifts. These effects are linked to the significant buildup of the T state, which intensifies with increased bromination. The effect of $I_{0}$ on ${\left\langle F\right\rangle }_{\mathrm{norm}}$ signals of CFl‐1Br and CFl‐2Br molecules were additionally examined under varying flow rates, and the same trends seen for CFl and CFl‐4Br were observed for these dyes (see Figures S2 and S3). Overall, all computational results demonstrate that increasing the number of Br atoms gives rise to more pronounced changes in ${\left\langle F\right\rangle }_{\mathrm{norm}}$ signals, particularly at higher flow speeds. This highlights the utility of our proposed method in detecting halogenation effects as well as resolving redox state dynamics under varying flow conditions.

**FIGURE 8:**
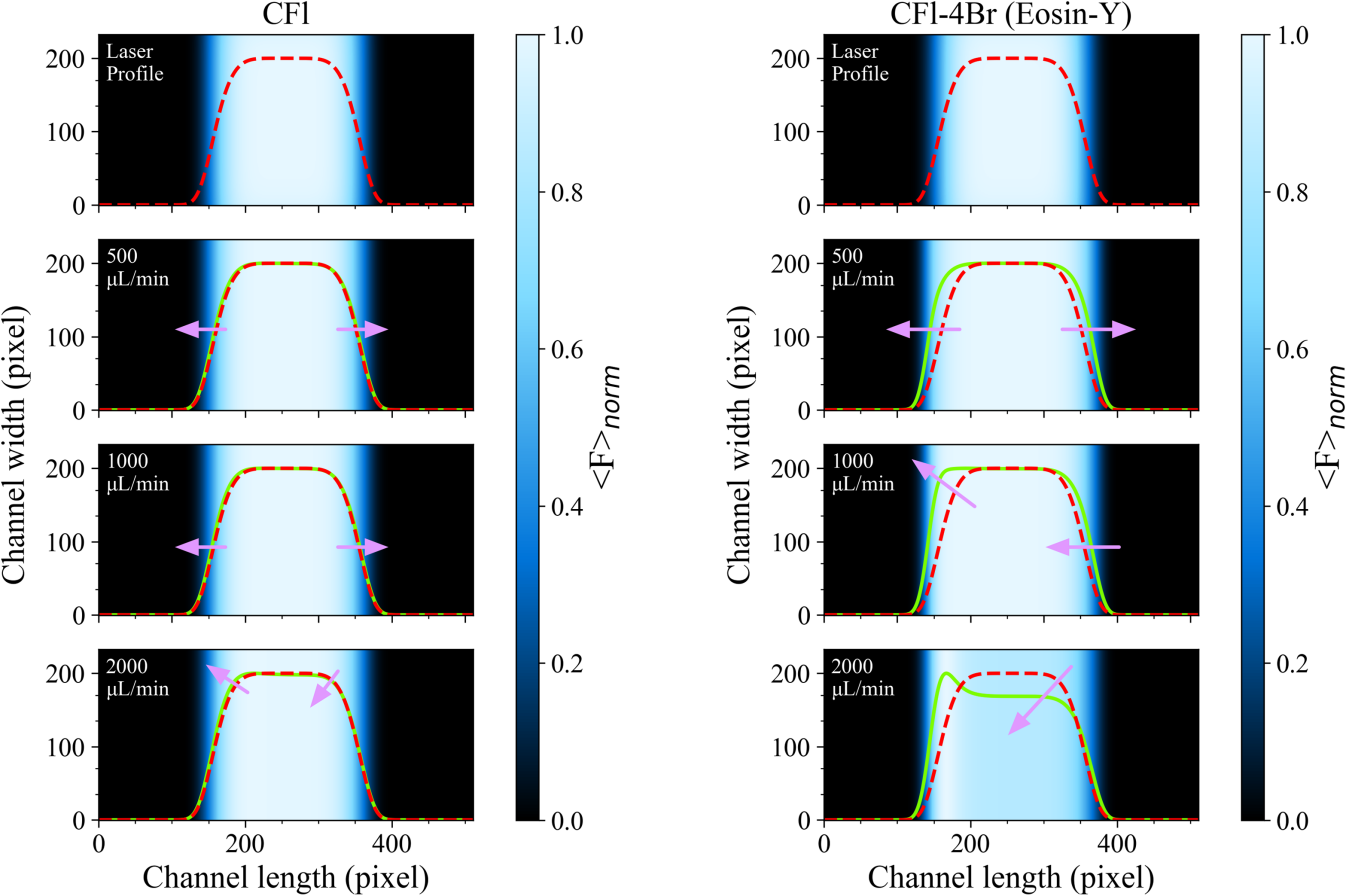
${\left\langle F\right\rangle }_{\mathrm{norm}}$ images computed for CFl (left) and CFl‐4Br (right) at different flow rates in microfluidics under a constant excitation irradiance of 3.62 kW/cm^2^ in which both fluorophores display the highest dark‐transient build‐ups during their passage over top flat beam area. Concomitant reduction in signal amplitudes of normalized image data simulated for CFl‐4Br molecules shows the trackability of internal heavy atom effect of Br atoms when compared to CFl. Color bars given on the right show the alterations in ${\left\langle F\right\rangle }_{\mathrm{norm}}$ signal when fluorophores pass over excitation beam. Dashed red signal shows the laser beam profile (that has no dark state build‐up) while green solid signal line represents the ${\left\langle F\right\rangle }_{\mathrm{norm}}$ signal averaged from the image data.

In addition to ${\left\langle F\right\rangle }_{\mathrm{norm}}$ signals computed for fluorescent dyes by using their electronic transition rates acquired from experiment‐based studies, one can also project the results of numerical simulations onto 2D arrays and generate normalized fluorescence image data which can be potentially captured through a sCMOS camera when it comes to perform a real measurement on fluorophores using an experimental optical setup. As the parameters including $q_{D}$ and $q_{F}$ can display differences based on the specifications of sCMOS camera and fluorophores used in experiments, fluorescence intensity recorded from samples would be different. For that reason, normalized fluorescence signal (${\left\langle F\right\rangle }_{\mathrm{norm}}$) images were simulated as most experimental parameters can be disregarded thanks to the normalization process. ${\left\langle F\right\rangle }_{\mathrm{norm}}$ images that are anticipated at different flow rates under a constant $I_{0}$ of 3.62 kW/cm^2^ for both CFl and CFl‐4Br were projected onto 2D grids and presented in Figure 8. Additionally, grayscale images, that consistent with those typically captured by sCMOS cameras, were generated and presented in Figure S4 (see supplementary file). Simulated images showed an ostensible broadening particularly for CFl‐4Br molecules in contrast to CFl due to the presence of four heavy Br atoms that remarkably promotes the populations of long‐lived T states particularly at high flow rates. Upon a gradual increment in flow rates up to 2000 μL/min, the population of long‐lived T state became more pronounced exclusively for CFl‐4Br owing to an enhanced spin‐orbit coupling. The level of normalized intensity gets dimmer in the pixels of simulated images along the direction of flow when fluorophores pass over the excitation beam area. There is no increment observed in the signal over uniform excitation area, also demonstrating that fluorophores cannot recover back to S state as they undergo T and ${\dot{R}}^{+}\left(t\right)$ states. However, these populations decrease when fluorophores leave the excitation beam area. This prominent, continual impairment in ${\left\langle F\right\rangle }_{\mathrm{norm}}$ amplitude displays how fluorophores residing in dark transient states can diminish the overall fluorescence of the fluorophore by promoting transitions to nonfluorescent states to a great extent. By carefully adjusting the flow rate in microfluidic channels, the residence time of fluorophores in dark transient states can be modulated, providing a potential means to control the photophysical behavior of fluorophores containing heavy atoms like Br.

## 5 Conclusions

Research findings presented in this study outlines how long‐lived, dark transient states of brominated CFl emitters, where Br containing derivatives have relatively higher triplet quantum yields and emit photons almost in the same spectral regions (510–670 nm), can be resolved by a proposed microfluidics‐based fluorescence microscope that can be implemented on optical table. Given the knowledge of electronic transition rates, fluorescence lifetimes of fluorophores, optical, geometrical and flow parameters of experimental system, ${\left\langle F\right\rangle }_{\mathrm{norm}}$ signals were simulated numerically for studied molecules when they flow over uniform excitation beam with top flat laser profile. Under varying excitation irradiances of laser beam and different flow rates within a microfluidic platform, it was inferred that populations of T and ${\dot{R}}^{+}\left(t\right)$ states of these molecules could be monitored since relative changes in simulated ${\left\langle F\right\rangle }_{\mathrm{norm}}$ signals appeared very discretely. Upon continual excitation, dark state populations manifested as signal broadening, shifts and decays in computed ${\left\langle F\right\rangle }_{\mathrm{norm}}$ signals that were derived from simulated fluorescence images. Notably, these effects were found to become more pronounced when the degree of bromination increases on CFl dye, showcasing the successful resolution of internal heavy atom effect by optimizing excitation power of laser and flow rates. Our proposed microfluidics‐based imaging methodology offers seamless, solid measurements since there is a reduced risk of photobleaching for fluorophores where they travel along a continuous excitation beam only once. The method capitalizes mainly on transition rates of dark transient states, and numerical simulations can thus be adapted to different types of fluorescent markers such as cyanine‐based dyes that can undergo trans‐cis isomerization reflecting subtle changes in viscosity of local environment and folding states of macromolecules. Our approach brings fluorescence‐based imaging one step closer to the conventional applications of microfluidics and traditional flowmetry studies. From a biomedical standpoint, monitoring fluorophore blinking of fluorophores that are tagged on biological entities such as exosomes, vesicles or specific organelles in cells, with our proposed method will offer new avenues in clinical studies. The proposed method has the potential to be used as a transient‐state sensor capable of differentiating healthy cells from various cancer cells that consume less oxygen compared to healthy cells (Warburg effect) [39], since oxygen concentration directly influences the populations of triplet state.

## Author Contributions

**Selim Can Dirican:** methodology, software, formal analysis, writing – review, editing. **Barış Demirbay:** visualization, conceptualization, methodology, software, formal analysis, writing – original draft, writing – review, editing and funding.

## Conflicts of Interest

The authors declare no conflicts of interest.

## Supporting information


**Figure S1.** The effect of beam size on ${\left\langle F\right\rangle }_{\mathrm{norm}}$ signals simulated for carboxyfluorescein (CFl) molecules flowing under constant flow rates of 2000 μL/min at varying optical power intensities: **(a)** w=30, **(b)** w=40, and **(c)** w=50μm, respectively. ${\left\langle F\right\rangle }_{\mathrm{norm}}$ signals were magnified in the ${\left\langle F\right\rangle }_{\mathrm{norm}}$ range between 1 and 0.94 to display the effect of the same beam sizes that are **(d)** w=30, **(e)** w=40, and **(f)** w=50μm, respectively. $I_{0}$ values computed for excitation beams having different w sizes are listed in Table S2.

**FIGURE S2.** The influence of excitation irradiance, $I_{0}$ varying from 0.06 to 3.62 kW/cm^2^ on ${\left\langle F\right\rangle }_{\mathrm{norm}}$ signals of monobromo‐carboxyfluorescein (CFl‐1Br) molecules flowing under constant flow rates of **(a)** 100, **(b)** 500, **(c)** 1000, and **(d)** 2000 μL/min, respectively. In figures, passage times of fluorophores over excitation beam computed for different flow rates were given in lower x‐axis, colorbar shows $I_{0}$ values and dashed black line shows the normalized beam profile signal (that has no dark state build‐up) as presented in Figure 4b.

**Figure S3.** The influence of excitation irradiance, $I_{0}$ varying from 0.06 to 3.62 kW/cm^2^ on ${\left\langle F\right\rangle }_{\mathrm{norm}}$ signals of dibromo‐carboxyfluorescein (CFl‐2Br) molecules flowing under constant flow rates of **(a)** 100, **(b)** 500, **(c)** 1000, and **(d)** 2000 μL/min, respectively. In figures, passage times of fluorophores over excitation beam computed for different flow rates were given in lower x‐axis, colorbar shows $I_{0}$ values and dashed black line shows the normalized beam profile signal (that has no dark state build‐up) as presented in Figure 4b

**Figure S4.** ${\left\langle F\right\rangle }_{\mathrm{norm}}$ images (a standard grayscale color map used in sCMOS cameras) computed for CFl (left) and CFl‐4Br (right) at different flow rates in microfluidics under a constant excitation irradiance of 3.62 kW/cm^2^ in which both fluorophores are expected to display the highest dark‐transient build‐ups during their uniform, laminar flow. Pink arrows show how dark transient state build‐ups change the shape of signals. The colorbars given on the right shows the alterations in ${\left\langle F\right\rangle }_{\mathrm{norm}}$ signal as fluorophores pass over excitation beam. Dashed red signal shows the laser beam profile (that has no dark state build‐up) while green solid signal line represents the ${\left\langle F\right\rangle }_{\mathrm{norm}}$ signal averaged from the image data.

**Table S1.** List of simulation parameters used in image data produced for fluorophores.
**Table S2** $I_{0}$ values calculated for laser excitation beams having different beam waist radius (w) values at different laser output powers.

## Data Availability

Custom‐made python script developed to simulate research findings outlined in the present work can be made available by the authors upon a reasonable request.
