# Supplementary figures and images for: A Novel Microfluidic‐Based Fluorescence Detection Method Reveals Heavy Atom Effects on Photophysics of Fluorophores With High Triplet Quantum Yield: A Numerical Simulation Study
Source: Luminescence. 2025 Jan 20;40(1):e70090. doi: 10.1002/bio.70090 (PMC11745564; doi:10.1002/bio.70090)

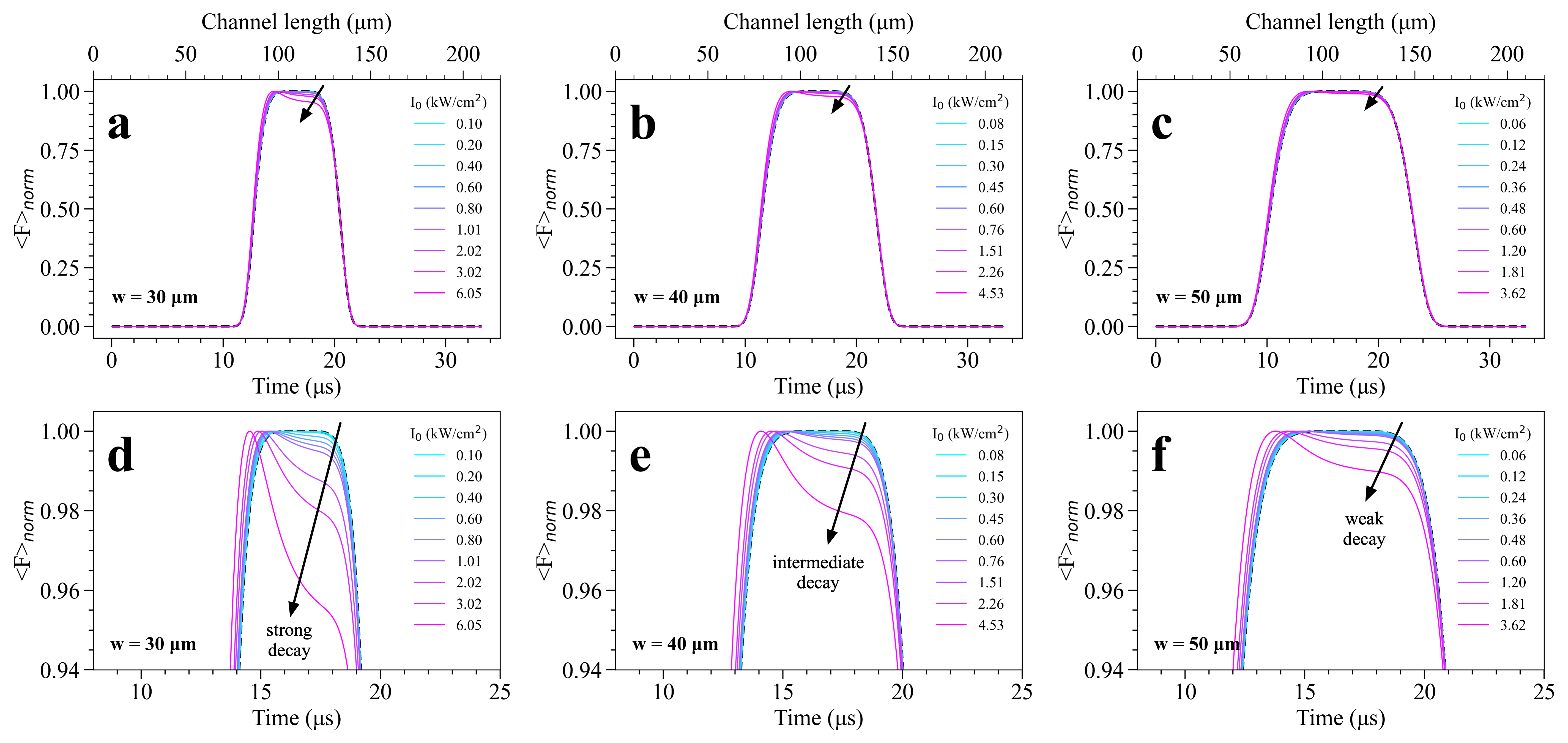

Supplement: Supplementary file 1 — Figure S1. The effect of beam size on Fnorm signals simulated for carboxyfluorescein (CFl) molecules flowing under constant flow rates of 2000 μL/min at varying optical power intensities: (a) w=30, (b) w=40, and (c) w=50μm, respectively. Fnorm signals were magnified in the Fnorm range between 1 and 0.94 to display the effect of the same beam sizes that are (d) w=30, (e) w=40, and (f) w=50μm, respectively. I0 values computed for excitation beams having different w sizes are listed in Table S2. [file BIO-40-e70090-s004.png]

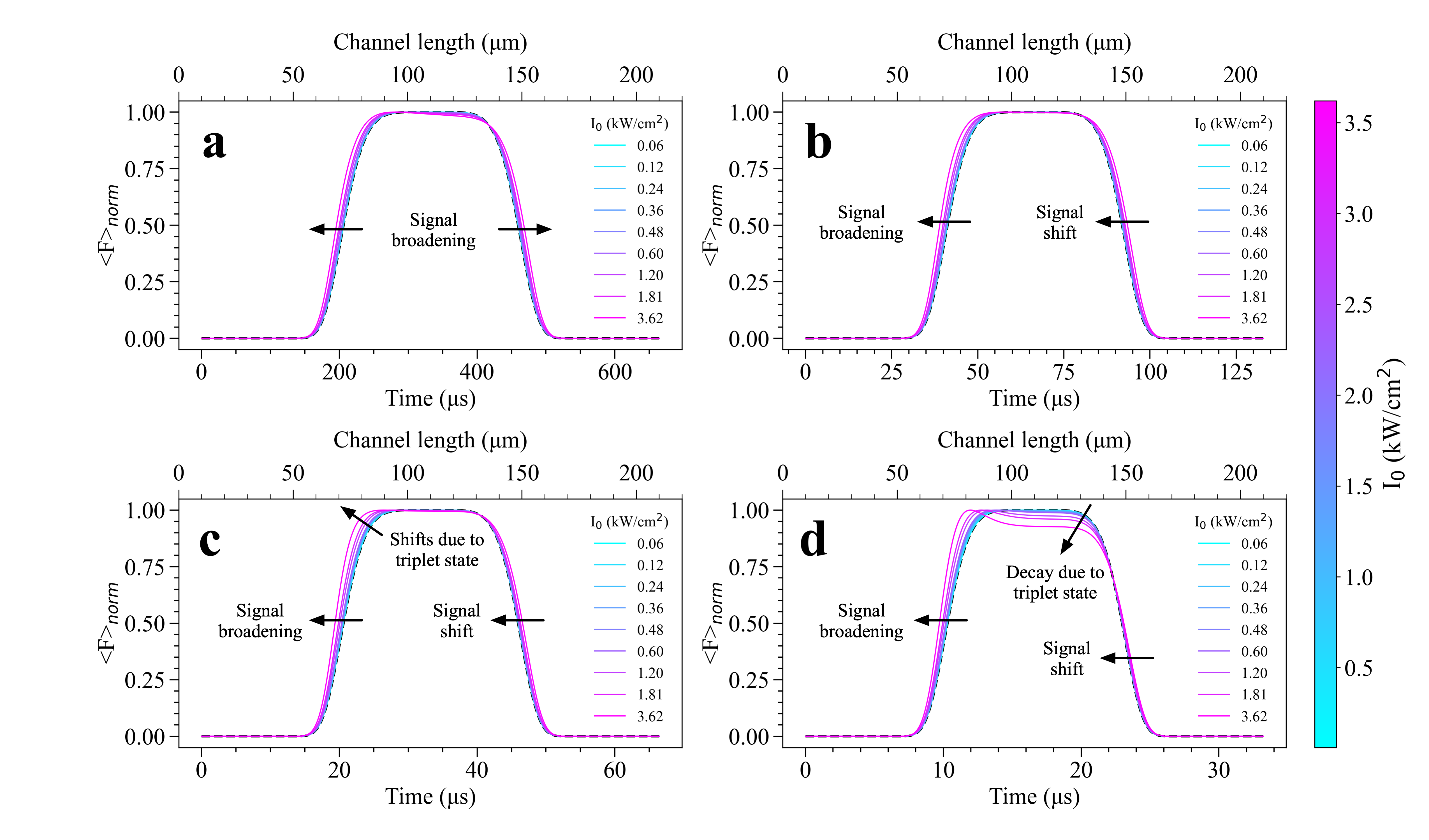

Supplement: Supplementary file 2 — FIGURE S2. The influence of excitation irradiance, I0 varying from 0.06 to 3.62 kW/cm2 on Fnorm signals of monobromo‐carboxyfluorescein (CFl‐1Br) molecules flowing under constant flow rates of (a) 100, (b) 500, (c) 1000, and (d) 2000 μL/min, respectively. In figures, passage times of fluorophores over excitation beam computed for different flow rates were given in lower x‐axis, colorbar shows I0 values and dashed black line shows the normalized beam profile signal (that has no dark state build‐up) as presented in Figure 4b. [file BIO-40-e70090-s005.png]

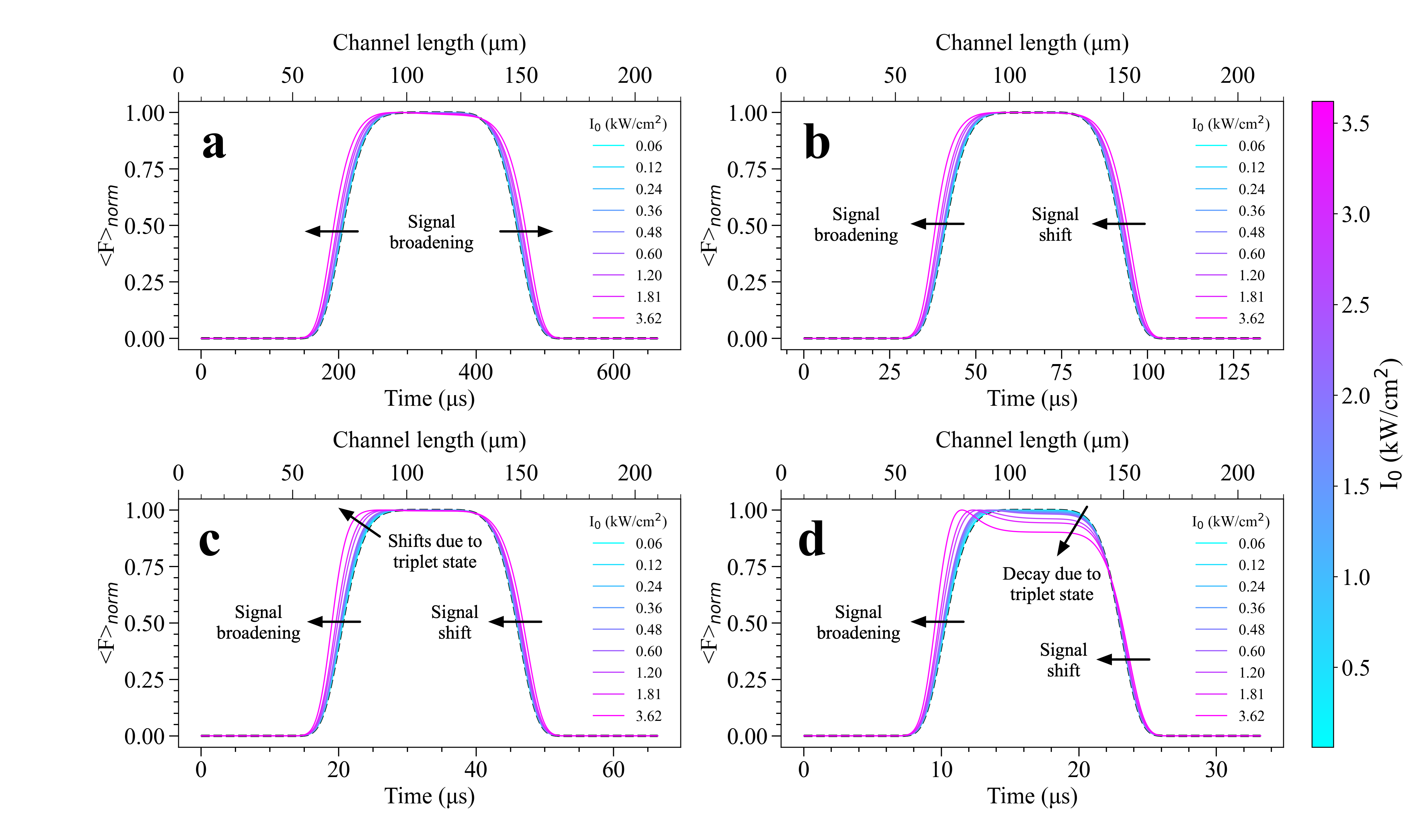

Supplement: Supplementary file 3 — Figure S3. The influence of excitation irradiance, I0 varying from 0.06 to 3.62 kW/cm2 on Fnorm signals of dibromo‐carboxyfluorescein (CFl‐2Br) molecules flowing under constant flow rates of (a) 100, (b) 500, (c) 1000, and (d) 2000 μL/min, respectively. In figures, passage times of fluorophores over excitation beam computed for different flow rates were given in lower x‐axis, colorbar shows I0 values and dashed black line shows the normalized beam profile signal (that has no dark state build‐up) as presented in Figure 4b [file BIO-40-e70090-s001.png]

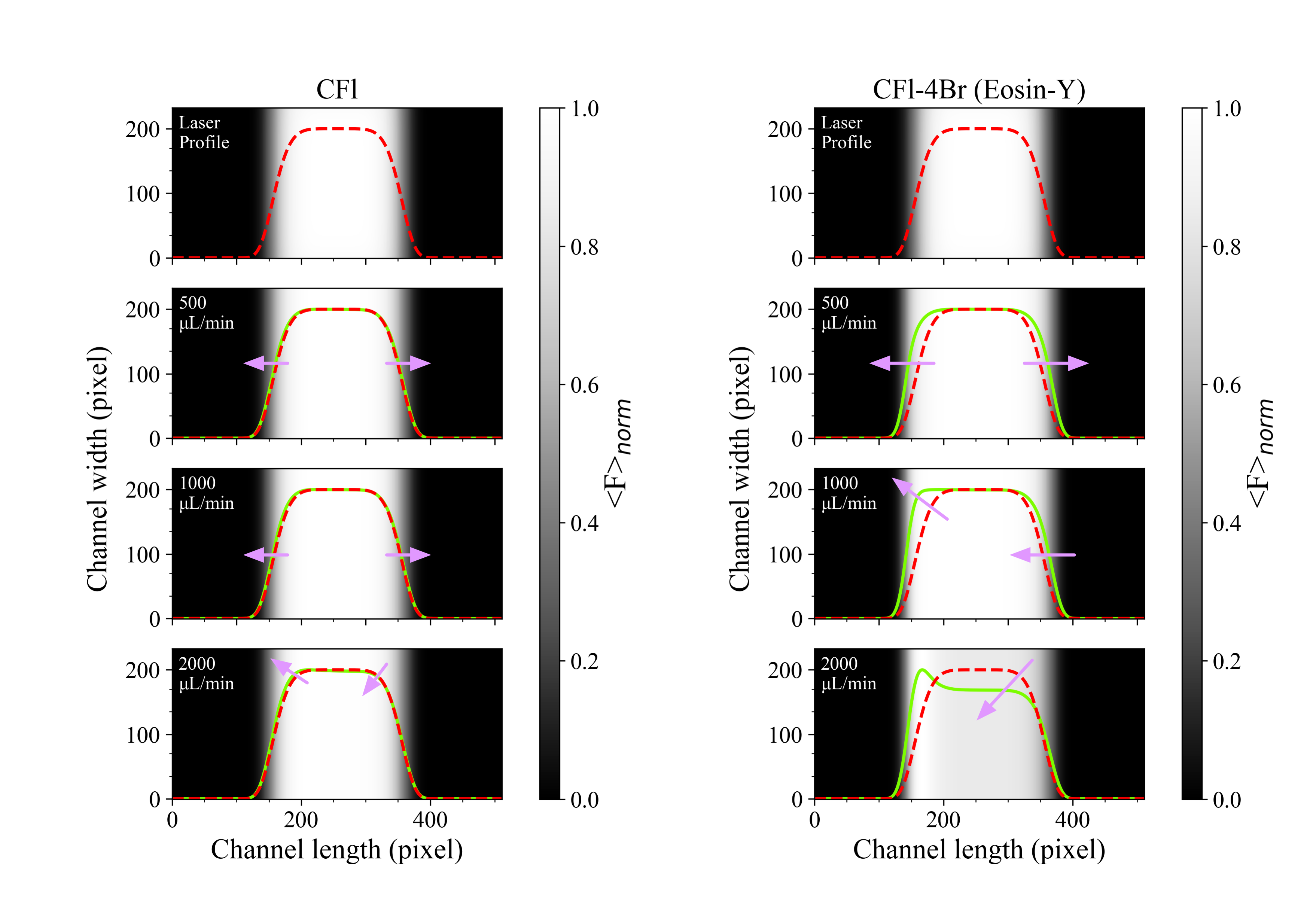

Supplement: Supplementary file 4 — Figure S4. Fnorm images (a standard grayscale color map used in sCMOS cameras) computed for CFl (left) and CFl‐4Br (right) at different flow rates in microfluidics under a constant excitation irradiance of 3.62 kW/cm2 in which both fluorophores are expected to display the highest dark‐transient build‐ups during their uniform, laminar flow. Pink arrows show how dark transient state build‐ups change the shape of signals. The colorbars given on the right shows the alterations in Fnorm signal as fluorophores pass over excitation beam. Dashed red signal shows the laser beam profile (that has no dark state build‐up) while green solid signal line represents the Fnorm signal averaged from the image data. [file BIO-40-e70090-s002.png]
