# Supplementary material for: A Novel Microfluidic‐Based Fluorescence Detection Method Reveals Heavy Atom Effects on Photophysics of Fluorophores With High Triplet Quantum Yield: A Numerical Simulation Study
Source: Luminescence. 2025 Jan 20;40(1):e70090. doi: 10.1002/bio.70090 (PMC11745564; doi:10.1002/bio.70090)
Supplement: Supplementary file 5 — Table S1. List of simulation parameters used in image data produced for fluorophores. Table S2 I0 values calculated for laser excitation beams having different beam waist radius (w) values at different laser output powers. [file BIO-40-e70090-s003.docx]

**Supplementary Information**

A novel microfluidic-based fluorescence detection method reveals heavy atom effects on photophysics of fluorophores with high triplet quantum yield: A numerical simulation study

Selim Can Dirican^1^, Barış Demirbay^2,*^

^1^Department of Mechanical Engineering, Özyeğin University, Istanbul, 34794, Türkiye

^2^Department of Mathematical and Natural Sciences, Özyeğin University, Istanbul, 34794, Türkiye

*Corresponding Author: Barış Demirbay

E-mail: baris.demirbay@ozyegin.edu.tr

Office phone: +902165649140

**Section 1.** **Details regarding computational data and simulation parameters**

Numerical calculations and modeling studies presented in this research were carried out by using different digital libraries, including numpy, matplotlib, scipy and so forth, in Jupyter Anaconda, Python environment. 2 dimensional grid, e.g. 2 arrays constructed separately for x and y dimensions, are employed to simulate the image data where both excitation area of laser beam and normalized fluorescence signal images are produced. For a standard sCMOS camera system, image data contains 2048 square pixels in horizontal and vertical dimensions and one square pixel size is 6.5 µm in length. In order to obtain a large amount of image data containing many frames during measurements on optical setup, the total number of pixels in both dimensions can be converted from 2048 to 512 pixels via 4x4 binning, which allows the sCMOS camera to record the frames much faster. Therefore, the horizontal (channel length) and vertical dimensions (channel width) of the images produced in the present study consist of 512 and 233 square pixels. Thereby, the total channel length and channel width are measured to be 221.86 µm and 100 µm, respectively. Using the supergaussian function given by Eq. 5 in the main text, the flat-top laser beam profile stretched across the flow direction was created using the following parameters: $I_{0}$ = 5000 a.u., w = 50 µm, L = 100 µm, x_0_ = 110.93 µm, y_0_ = 50 µm and n = 6, so that the beam profile is produced in the center coordinates of produced images and presented by heatmaps with color bars in Figure 4. Different w values tested in complementary signal simulations are presented in Figure S1. To create laser excitation beam profile in produced images, it is necessary to process I(x,y) signal for each single pixel in the image data by using “for loop”. Starting with the I(x,0) signal, that is I values at each pixel along the channel length (the same direction as laminar flow), corresponding to y = 0, were simulated using Equation 5. The same simulations were then performed using “for loop” for the rest of 232 pixels along channel width direction until the final value of y = 233 was reached, and the I(x,y) values for each x and y coordinate were simulated one by one. Each computed I(x,y) value were assigned to different pixel pairs in camera image that have a total area of 512 x 233 pixel^2^. To simulate the fluorescence intensities (using analytical solution of photophysical model) in each single pixel, $k_{01}$ values need to be simulated on these pixels. Starting with the first pixel in vertical dimension (along channel width), corresponding to y=0, the I(t=x/v,0) signal, that is the time-dependent fluorescence intensity signal, is generated along the chip length consisting of 512 pixels. This process is repeated for the rest of 232 pixels along the channel width until the final pixel value y = 233 is reached. Considering this process, $\left\langle F(x,y) \right\rangle$ values were calculated for each pixel for all x and y coordinates and then converted to time averaged fluorescence signal in the direction of laminar flow to show the effects of dark transient state build-ups. Complete list of all simulation parameters were tabulated in Table S1.

**Table S1.** List of simulation parameters used in image data produced for fluorophores.

| Simulation parameters with units | Values / Range |
| --- | --- |
| Laser wavelength (nm) | 488 |
| Laser output power (mW) | 5 – 300 |
| Magnitude of laser profile intensity (a.u.) | 5000 |
| Magnification factor of microscope objective | 60x |
| Binning factor of sCMOS camera | 4 x 4 |
| Pixel size of sCMOS camera ($\mu$m) | 6.5 |
| Horizontal dimension of $\left\langle F \right\rangle_{\mathrm{norm}}$ images (pixel) | 512 |
| Vertical dimension of $\left\langle F \right\rangle_{\mathrm{norm}}$ images (pixel) | 233 |
| Total area of $\left\langle F \right\rangle_{\mathrm{norm}}$ images (pixel^2^) | 512 x 233 |
| Pixel size on sample plane ($\mu$m/pixel) | 0.43 |
| Horizontal length of $\left\langle F \right\rangle_{\mathrm{norm}}$ images ($\mu$m) | 221.86 |
| Vertical length of $\left\langle F \right\rangle_{\mathrm{norm}}$ images ($\mu$m) | 100 |
| Vertical length of excitation beam, L ($\mu$m) | 100 |
| Beam waist radius (along x-axis) of excitation laser, $w$ ($\mu$m) | 30 – 50 |
| Order of supergaussian beam profile, n | 6 |
| Flow rate ($\mu L$/min) | 100 – 2000 |
| Channel height of microfluidics chip ($\mu$m) | 50 |
| Channel width of microfluidics chip ($\mu$m) | 100 |
| Channel length of microfluidics chip ($\mu$m) | 221.86 |

**Section 2.** **Complementary simulation results**


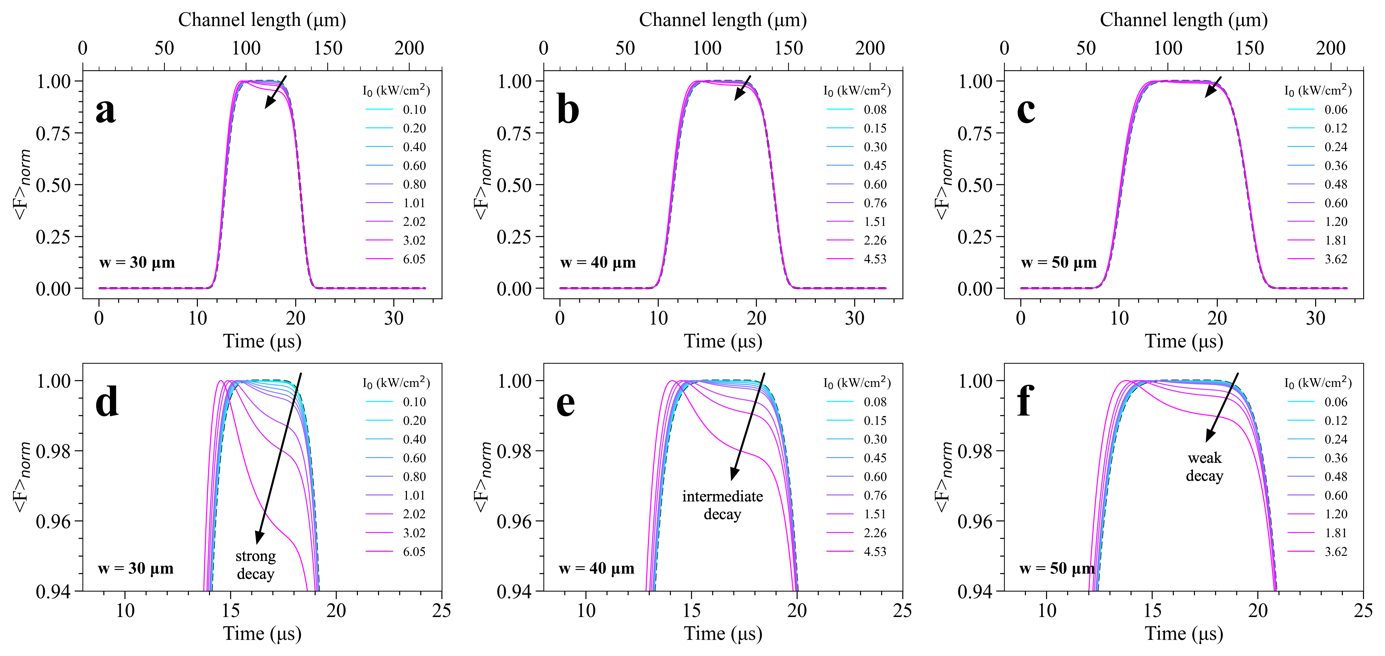


**Figure S1.** The effect of beam size on $\left\langle F \right\rangle_{\mathrm{norm}}$ signals simulated for carboxyfluorescein (CFl) molecules flowing under constant flow rates of 2000 $\mu$L/min at varying optical power intensities: **(a)**$w=30 \mu$m, **(b)**$w=40 \mu$m and **(c)**$w=50 \mu$m, respectively. $\left\langle F \right\rangle_{\mathrm{norm}}$ signals were magnified in the $\left\langle F \right\rangle_{\mathrm{norm}}$ range between 1 and 0.94 to display the effect of the same beam sizes that are **(d)**$w=30 \mu$m, **(e)**$w=40 \mu$m, **(f)**$w=50 \mu$m, respectively. $I_{0}$ values computed for excitation beams having different $w$ sizes are listed in Table S2.

**Table S2.** $I_{0}$ values calculated for laser excitation beams having different beam waist radius ($w$) values at different laser output powers.

| Laser output  powers (mW) | Excitation irradiance, $I_{0}$ (kW/cm^2^) | | |
| --- | --- | --- | --- |
|  | $w$ = 30 $\mu$m | $w$ = 40 $\mu$m | $w$ = 50 $\mu$m |
| 5 | 0.10 | 0.08 | 0.06 |
| 10 | 0.20 | 0.15 | 0.12 |
| 20 | 0.40 | 0.30 | 0.24 |
| 30 | 0.60 | 0.45 | 0.36 |
| 40 | 0.80 | 0.60 | 0.48 |
| 50 | 1.01 | 0.76 | 0.60 |
| 100 | 2.02 | 1.51 | 1.20 |
| 150 | 3.02 | 2.26 | 1.81 |
| 300 | 6.05 | 4.53 | 3.62 |


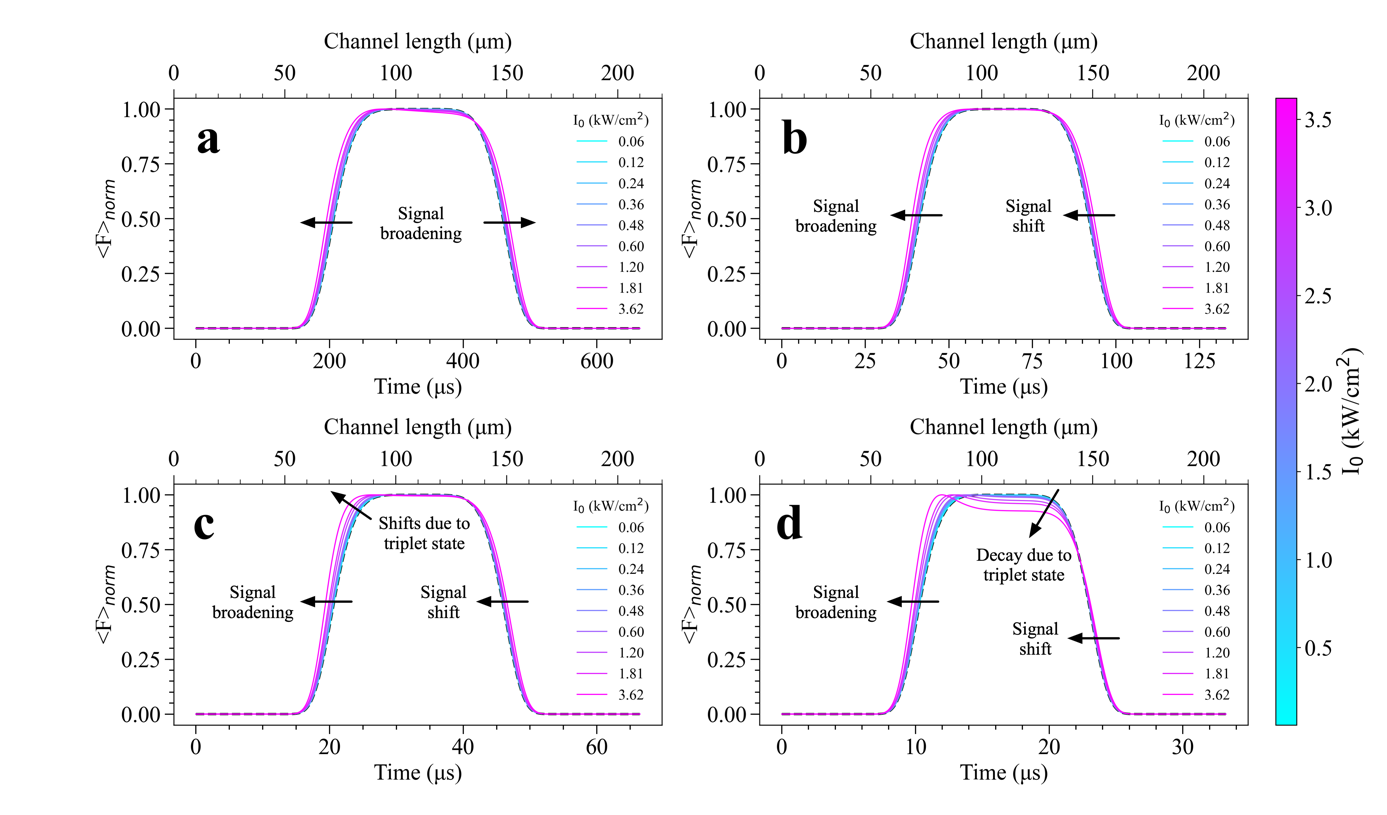


**Figure S2.** The influence of excitation irradiance, $I_{0}$ varying from 0.06 to 3.62 kW/cm^2^ on $\left\langle F \right\rangle_{\mathrm{norm}}$ signals of mono-bromo-carboxyfluorescein (CFl-1Br) molecules flowing under constant flow rates of **(a)** 100 $\mu$L/min, **(b)** 500 $\mu$L/min, **(c)** 1000 $\mu$L/min and **(d)** 2000 $\mu$L/min, respectively. In figures, passage times of fluorophores over excitation beam computed for different flow rates were given in lower x-axis, colorbar shows $I_{0}$ values and dashed black line shows the normalized beam profile signal (that has no dark state build-up) as presented in Figure 4(b).


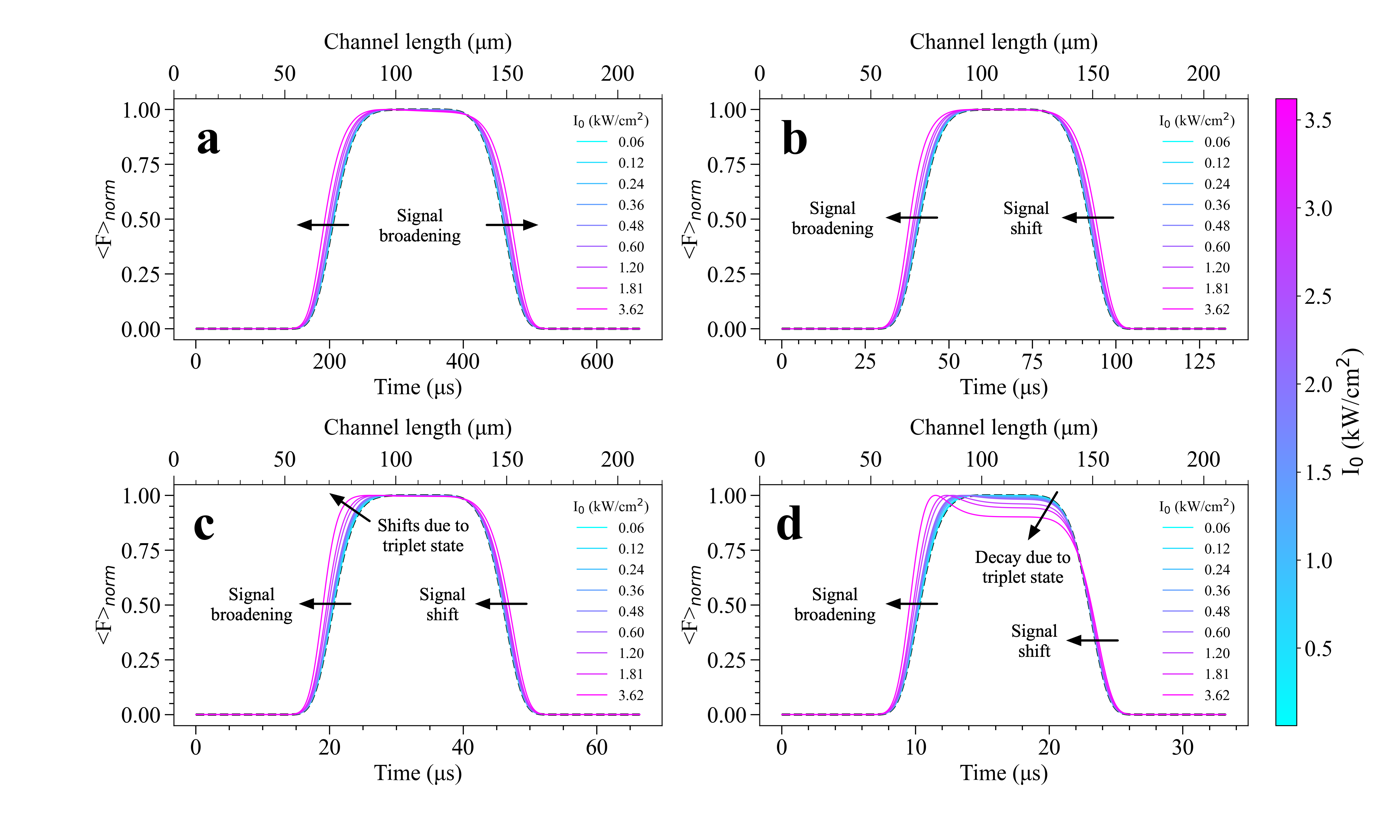


**Figure S3.** The influence of excitation irradiance, $I_{0}$ varying from 0.06 to 3.62 kW/cm^2^ on $\left\langle F \right\rangle_{\mathrm{norm}}$ signals of di-bromo-carboxyfluorescein (CFl-2Br) molecules flowing under constant flow rates of **(a)** 100 $\mu$L/min, **(b)** 500 $\mu$L/min, **(c)** 1000 $\mu$L/min and **(d)** 2000 $\mu$L/min, respectively. In figures, passage times of fluorophores over excitation beam computed for different flow rates were given in lower x-axis, colorbar shows $I_{0}$ values and dashed black line shows the normalized beam profile signal (that has no dark state build-up) as presented in Figure 4(b).


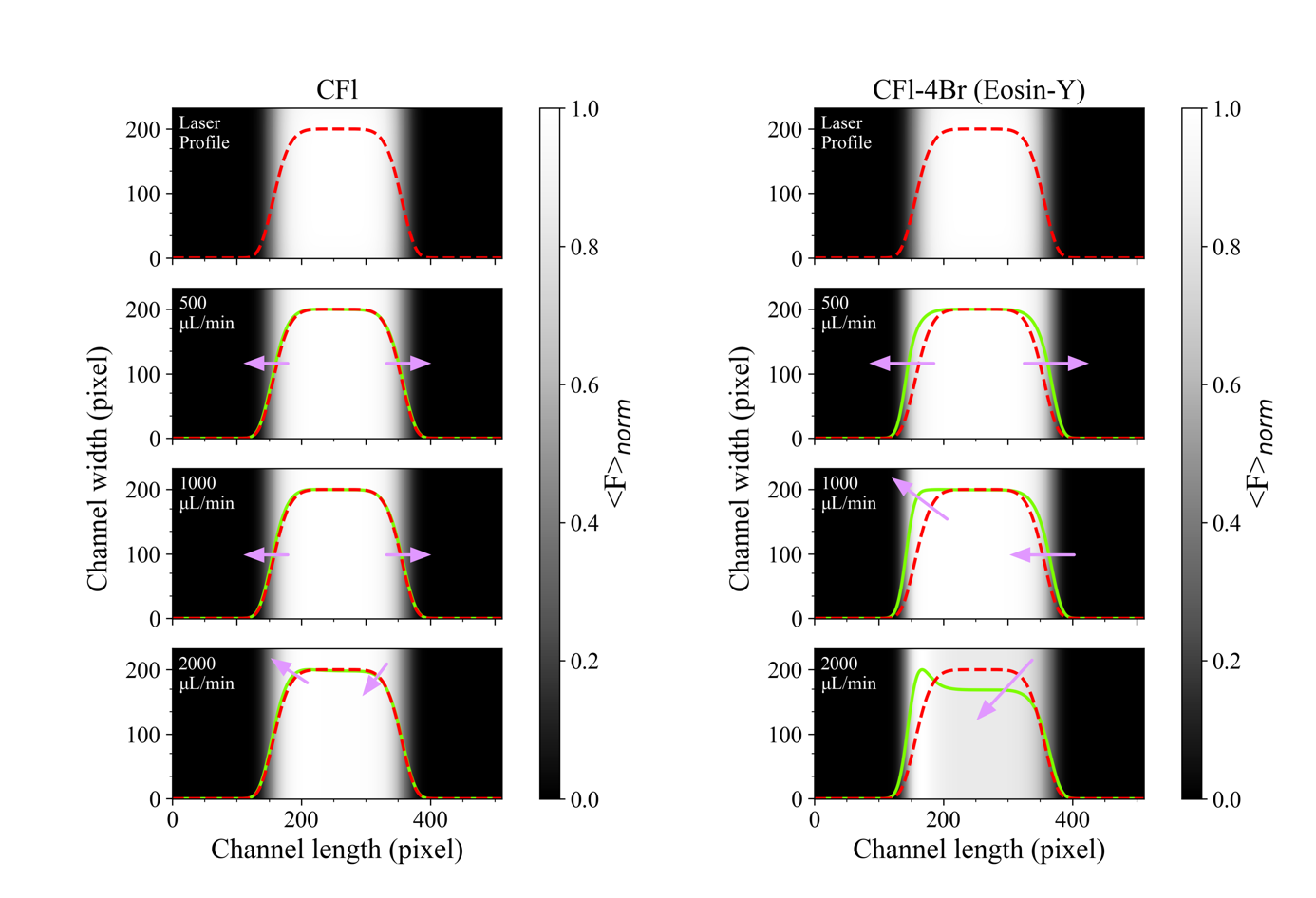


**Figure S4.** $\left\langle F \right\rangle_{\mathrm{norm}}$ images (a standard grayscale color map used in sCMOS cameras) computed for CFl (left) and CFl-4Br (right) at different flow rates in microfluidics under a constant excitation irradiance of 3.62 kW/cm^2^ in which both fluorophores are expected to display the highest dark-transient build-ups during their uniform, laminar flow. Pink arrows show how dark transient state build-ups change the shape of signals. Colorbars given on the right shows the alterations in $\left\langle F \right\rangle_{\mathrm{norm}}$ signal when fluorophores pass over excitation beam. Dashed red signal shows the laser beam profile (that has no dark state build-up) while green solid signal line represents the $\left\langle F \right\rangle_{\mathrm{norm}}$ signal averaged from the image data.
